# Supplementary material for: Recursive Ectopic Gene Conversion Leads to Elevated DNA Mutation, Gene Loss, and Novel Gene Formation in Aspergillus
Source: Microorganisms. 2025 Dec 22;14(1):33. doi: 10.3390/microorganisms14010033 (PMC12844118; doi:10.3390/microorganisms14010033)

Supplemental Figures

Supplemental Figure S1. Exon Sequence Alignment Analysis of AF13 and RIB40 Strains.

This figure shows the exon sequence alignment results of four groups of homologous genes in AF13 and RIB40 strains. The corresponding relationships of genes in each group are as follows: A red background indicates that the four bases of two different species are identical. A green background indicates that three bases are the same. A gray background indicates that two bases are identical. a. The first group of genes corresponds to two genes in AF13, namely AF_{13}1g00222 and AF_{13}1g00232, and RIB40 also corresponds to two genes, aor8g00868 and aor8g00878. b. The second group of genes corresponds to two genes in AF13, namely AF_{13}1g00221 and AF_{13}1g00233, and six genes in RIB40. They are aor8g00865, aor8g00866, aor8g00867, aor8g00879, aor8g00880, aor8g00881; c. The third group of genes corresponds to two genes in AF13, namely AF_{13}1g00218 and AF_{13}1g00240, and three genes in RIB40, namely aor8g00859, aor8g00860 and aor8g00861. d. The fourth gene group corresponds to two genes in AF13, namely AF_{13}1g00206 and AF_{13}1g00246, and RIB40 also corresponds to two genes, namely aor8g00853 and aor8g00889. The sequence alignment results intuitively present the nucleotide differences and conservation characteristics in the exon regions of homologous genes from different strains, providing a molecular basis for analyzing gene function differentiation and evolutionary relationships.

a

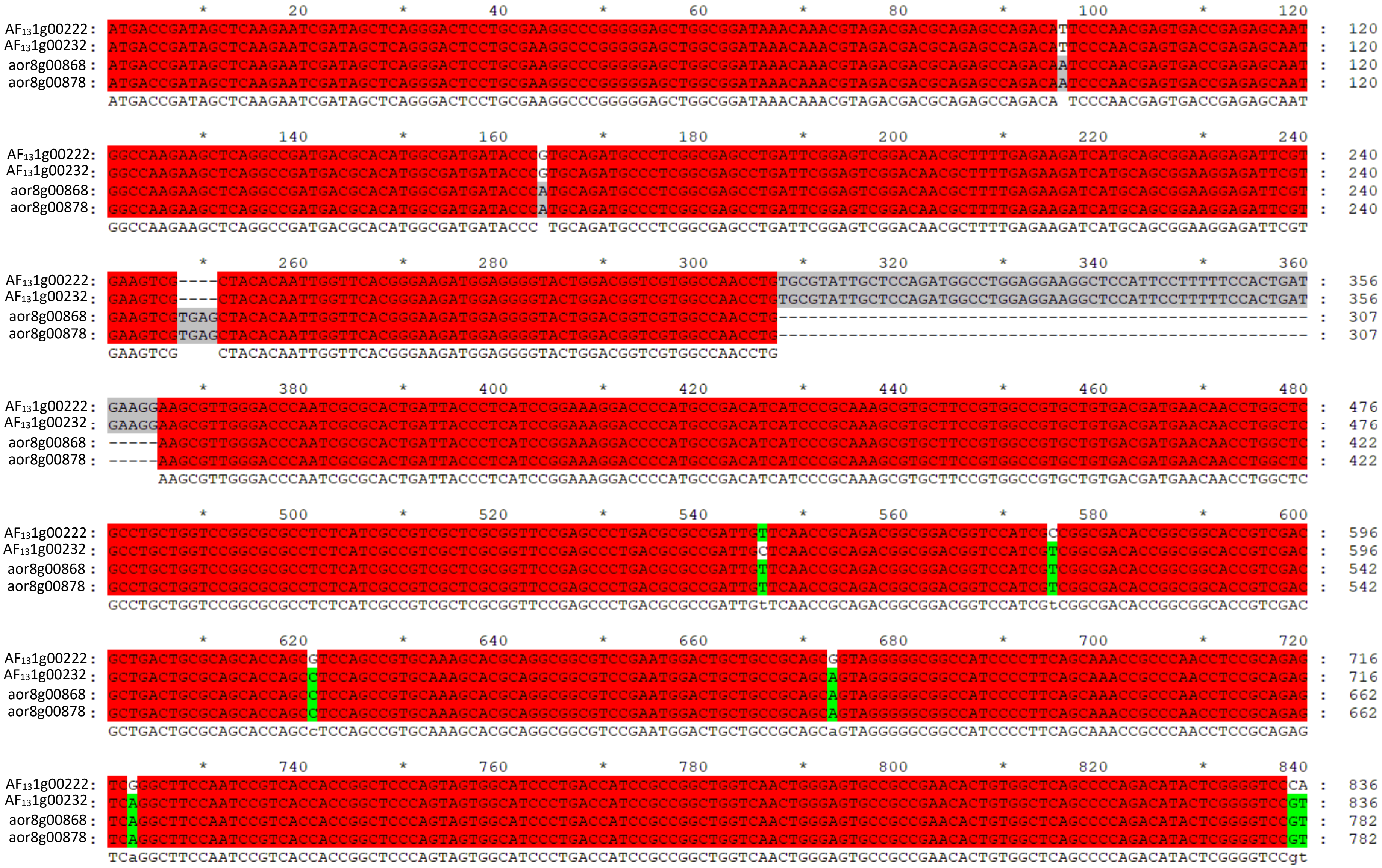

```
                    *       860         *       880         *       900         *       920         *       940         *       960
AF131g00222 : GACATACCAACCGCCGCCCACAACCGGTTTGACGACGGCTCCTGCGCCAGGCCCAGCAGGCCCAGCAGGGCCGCCCGCCCGCCCCCCGGTGCCCATGCCCACTGCATCCCAACCGCCACA :  956
AF131g00232 : GACATACCAACCGCCGCCCACAACCGGTTTGACGACGGCTCCTGCGCCAGGCCCAGCAGGCCCAGCAGGGCCGCCCGCCCGCCCCCCGGTGCCCATGCCCACTGCATCCCAACCGCCACA :  956
aor8g00868  : GACATACCAACCGCCGCCCACAACCGGTTTGACGACGGCTCCTGCGCCAGGCCCAGCAGGCCCAGCAGGGCCGCCCGCCCGCCCCCCGGTGCCCATGCCCACTGCATCCCAACCGCCACA :  902
aor8g00878  : GACATACCAACCGCCGCCCACAACCGGTTTGACGACGGCTCCTGCGCCAGGCCCAGCAGGCCCAGCAGGGCCGCCCGCCCGCCCCCCGGTGCCCATGCCCACTGCATCCCAACCGCCACA :  902
              GACATACCAACCGCCGCCCACAACCGGTTTGACGACGGCTCCTGCGCCAGGCCCAGCAGGCCCAGCAGGGCCGCCCGCCCGCCCCCCGGTGCCCATGCCCACTGCATCCCAACCGCCACA

                    *       980         *      1000         *      1020         *      1040         *      1060         *      1080
AF131g00222 : AGCACCGCTATGGACCACGGACGCTGTCCCGCACATTACCTCTGTTCAACCGCCGTCGGCGCCGGGAGTGGCGCCGGGGCCCTATCCCGGACAGTCCGTGAGTCACGGCGTGGCTGCGGG : 1076
AF131g00232 : AGCGCCGCTATGGACCGCGGATGCTGTCCCGCACATTACCTCAGTTCAACCGCCGTTGGCGCCGGGAGCGGCGCCGGGGCCCTATCCCGGACAGTCCGTGAGTCACGGCGTGGCTGCGGG : 1076
aor8g00868  : AGCGCCGCTATGGACCACGGATGCTGTCCCGCACATTACCTCAGTTCAACCGCCGTTGGCGCCGGGAGCGGCGCCGGGGCCCTATCCCGGACAGTCCGTGAGTCACGGCGTGGCTGCGGG : 1022
aor8g00878  : AGCGCCGCTATGGACCACGGATGCTGTCCCGCACATTACCTCAGTTCAACCGCCGTTGGCGCCGGGAGCGGCGCCGGGGCCCTATCCCGGACAGTCCGTGAGTCACGGCGTGGCTGCGGG : 1022
              AGCgCCGCTATGGACCaCGGAtGCTGTCCCGCACATTACCTCaGTTCAACCGCCGTtGGCGCCGGGAGcGGCGCCGGGGCCCTATCCCGGACAGTCCGTGAGTCACGGCGTGGCTGCGGG

                    *      1100         *      1120         *      1140         *      1160         *      1180         *      1200
AF131g00222 : CATGCCGCATCCCTCGCAGCCGCAGGTTAACCAGTCGATGCACCCTACCGCTCTCGCATACGGCTCGTTGCACCCGCAGGTGACTGGTCACCTTCCCTCCGGCCAGCTCTACGTCGGCCC : 1196
AF131g00232 : CATGCCGCATCCCTCGCAGCCGCAGGTTAACCAGTCGATGCACCCTACCGCTCTCGCATACGGCTCGTTGCACCCGCAGGTGACTGGTCACCTTCCCTCCGGCCAGCTCTACGCCGGCCC : 1196
aor8g00868  : CATGCCGCATCCCTCGCAGCCGCAGGTTAACCAGTCGATGCACCCTACCGCTCTCGCATACGGCTCGTTGCACCCGCAGGTGACTGGTCACCTTCCCTCCGGCCAGCTCTACGTCGGCCC : 1142
aor8g00878  : CATGCCGCATCCCTCGCAGCCGCAGGTTAACCAGTCGATGCACCCTACCGCTCTCGCATACGGCTCGTTGCACCCGCAGGTGACTGGTCACCTTCCCTCCGGCCAGCTCTACGTCGGCCC : 1142
              CATGCCGCATCCCTCGCAGCCGCAGGTTAACCAGTCGATGCACCCTACCGCTCTCGCATACGGCTCGTTGCACCCGCAGGTGACTGGTCACCTTCCCTCCGGCCAGCTCTACGtCGGCCC

                    *      1220         *      1240         *      1260         *      1280         *      1300         *      1320
AF131g00222 : AGCCGGTCAGGTTGTACAAGCTGGCCATGCTTTTCAAACCGGTCAGGCTGGCCAGGCTCTTCAAACTGGCCATGCTTTTCAAACCGGTCAGGCTGGCCAGGCTCTTCAAACTGGCCAAAC : 1316
AF131g00232 : AGCCGGTCAGGTTGTACAAGCGGGCCATGCTTTTCAAACCGGTCAGGCTGGTCAGGCTCTTCAAACTGGCCAAACTAGTCAAGTTGTCCAGACCGGTCAGGCTCTGCAAGCTGGTCAGGC : 1316
aor8g00868  : AGCCGGTCAGGTTGTACAAGCGGGCCATGCTTTTCAAACCGGTCAGGCTGGTCAGGCTCTTCAAACTGGCCAAACTAGTCAAGTTGTCCAGACCGGTCAGGCTCTTCAAGCTGGTCAGGC : 1262
aor8g00878  : AGCCGGTCAGGTTGTACAAGCGGGCCATGCTTTTCAAACCGGTCAGGCTGGTCAGGCTCTTCAAACTGGCCAAACTAGTCAAGTTGTCCAGACCGGTCAGGCTCTTCAAGCTGGTCAGGC : 1262
              AGCCGGTCAGGTTGTACAAGCgGGCCATGCTTTTCAAACCGGTCAGGCTGGtCAGGCTCTTCAAACTGGCCAaaCTagTCAAgttGtcCAGaCcGGtCAGGCTCTtCAAgCTGGtCAggC

                    *      1340         *      1360         *      1380         *      1400         *      1420         *      1440
AF131g00222 : TAGTCAAGTTGTCCAGACCGGTCAGGCTCTTCAAGCTGGTCAGGCCGTGCAAGCTGGTCAGACCGTTCAAACTGTTCAAGCTGGTCCGACTGCTCAAACTGCTCATACTGTTCAAGCTGT : 1436
AF131g00232 : TCTTCAAGCTG---------GTCAGGCTAGTCAAGCTGGTCAGGCTGTGCAAGCTGGTCAGACCGTTCAACCTGTTCAAGCTGGTCCGACTGCTCAAACTGCTCATACTGTTCAAGCTGT : 1427
aor8g00868  : TCTTCAAGCTG---------GTCAGGCTAGTCAAGCTGGTCAG------------------ACCGTTCAACCTGTTCAAGCTGGTCCGACTGCTCAAACTGCTCATACTGTTCAAGCTGT : 1355
aor8g00878  : TCTTCAAGCTG---------GTCAGGCTAGTCAAGCTGGTCAG------------------ACCGTTCAACCTGTTCAAGCTGGTCCGACTGCTCAAACTGCTCATACTGTTCAAGCTGT : 1355
              TctTCAAGcTG         GTCAGGCTagTCAAGCTGGTCAG                  ACCGTTCAAcCTGTTCAAGCTGGTCCGACTGCTCAAACTGCTCATACTGTTCAAGCTGT

                    *      1460         *      1480         *      1500         *      1520         *      1540         *      1560
AF131g00222 : GCAAGCTGGTCTAGGGGCACCACCGCAACCTTCGTTTCCGATGGCCCACGGCCAACCTGCCGCTGCGGTCCCAAACGGCGGCGGCTTGCTGACCGCACCCCAGCCCGTTCAGCACTGGAC : 1556
AF131g00232 : GCAAGCTGGTCTAGGGGCACCACCGCAACCTTCGTTTCCGATGGCCCACGGCCAACCTGCCGCTGCGGTCCCAAACGGCGGCGGCTTGCTGACCGCACCCCAGCCCGTTCAGCACTGGAC : 1547
aor8g00868  : GCAAGCTGGTCTAGGGGCACCACCGCAACCTTCGTTTCCGATGGCCCACGGCCAACCTGCCGCTGCGGTCCCAAACGGCGGCGGCTTGCTGACCGCACCCCAGCCCGTTCAGCACTGGAC : 1475
aor8g00878  : GCAAGCTGGTCTAGGGGCACCACCGCAACCTTCGTTTCCGATGGCCCACGGCCAACCTGCCGCTGCGGTCCCAAACGGCGGCGGCTTGCTGACCGCACCCCAGCCCGTTCAGCACTGGAC : 1475
              GCAAGCTGGTCTAGGGGCACCACCGCAACCTTCGTTTCCGATGGCCCACGGCCAACCTGCCGCTGCGGTCCCAAACGGCGGCGGCTTGCTGACCGCACCCCAGCCCGTTCAGCACTGGAC

                    *      1580         *      1600         *      1620         *      1640         *      1660         *      1680
AF131g00222 : GCCCGGCGTGGCGGGACTGCCGCAGGCAGCGACGCAAACCGCCCCTCCAACGCAACCGGTAAACGCCGCGCTGTCCGCCCAACCAACAGACCCGTGTCAGGGAACGCTGGTGCATCCTGG : 1676
AF131g00232 : GCCCGGCGTGGCGGGACTGCCGCAGGCAGCGACGCAAACCGCCCCTCCAACGCAACCGGTAAACGCCGCGCTGTCCGCCCAACCAACAGACCCGTGTCAGGGAACGCTGGTGCATCCTGG : 1667
aor8g00868  : GCCCGGCGTGGCGGGACTGCCGCAGGCAGCGACGCAAACCGCCCCTCCAACGCAACCGGTAAACGCCGCGCTGTCCGCCCAACCAACAGACCCGTGTCAGGGAACGCTGGTGCATCCTGG : 1595
aor8g00878  : GCCCGGCGTGGCGGGACTGCCGCAGGCAGCGACGCAAACCGCCCCTCCAACGCAACCGGTAAACGCCGCGCTGTCCGCCCAACCAACAGACCCGTGTCAGGGAACGCTGGTGCATCCTGG : 1595
              GCCCGGCGTGGCGGGACTGCCGCAGGCAGCGACGCAAACCGCCCCTCCAACGCAACCGGTAAACGCCGCGCTGTCCGCCCAACCAACAGACCCGTGTCAGGGAACGCTGGTGCATCCTGG
```

```
                     *      1700         *      1720         *      1740         *      1760         *      1780         *      1800
AF131g00222 : GCAGCCGCAGGCATCAACCCAACCGTCCACCGGGCACCCTCCCGTCGAATCATCTCAGCAAACACCCTGGCCACCCCAGCAGCAGAATGACTCTGCGATGGACAGTGACCCG-ATGGCCA : 1795
AF131g00232 : GCAGCCGCAGGCATCAACCCAACCGTCCACCGGGCACCCTCCCGTCGAACCATCTCAGCAAACACCCTGGCCACCCCAGCAGCAGAATGACTCTGCGATGGACAGTGACCCG-ATGGCCA : 1786
aor8g00868  : GCAGCCGCAGGCATCAACCCAACCGTCCACCGGGCACCCTCCCGTCGAACCATCTCAGCAAACACCCTGGCCACCCCAGCAGCAGAATGACTCTGCGATGGACAGTGACCCGTATGGGCT : 1715
aor8g00878  : GCAGCCGCAGGCATCAACCCAACCGTCCACCGGGCACCCTCCCGTCGAACCATCTCAGCAAACACCCTGGCCACCCCAGCAGCAGAATGACTCTGCGATGGACAGTGACCCGTATGGGCT : 1715
              GCAGCCGCAGGCATCAACCCAACCGTCCACCGGGCACCCTCCCGTCGAAcCATCTCAGCAAACACCCTGGCCACCCCAGCAGCAGAATGACTCTGCGATGGACAGTGACCCG ATGG C

                     *      1820         *      1840         *      1860         *      1880         *      1900         *      1920
AF131g00222 : GCGATGTCTCAGAGGATGCTCTGAACGCGGCCACGGAGGAAATCCGCCGACTGCAGGCGGACATGGCTCGCTATCGGGACGAGGTCGAGGAGCACATCCGATCCCTGAAACAGGCCCTGG : 1915
AF131g00232 : GCGATGTCTCAGAGGATGCTTTGAACGCGGCCACGGAGGAAATCCGCCGACTGCAAGCGGACATGGCTCGCTATCGGGATGAGGTCGAGGAGCACATCCGATCCCTGAAGCAGGCCCTGG : 1906
aor8g00868  : GAACCTTTT----GGATTCTTGGTCTCTATTCAAGTGA---------------------------------------------------------------------------------- : 1749
aor8g00878  : GAACCTTTT----GGATTCTTGGTCTCTATTCAAGTGA---------------------------------------------------------------------------------- : 1749
              G     T T    GGAT CTt G        CA G

                     *      1940         *      1960         *      1980         *      2000         *      2020         *      2040
AF131g00222 : ACGAGACCAATGAGCGGTACACAACCGAGATTCGGTACTATCCCCCGCCTCGGCCGAATGGTGCGGTCCGGGAGTCTCTGGGGCGACTATTGAATGCTGACCATGGTAACAGTGATATCC : 2035
AF131g00232 : ACGAGACCAATGAGCGGTACACAACCGAGATTCGGTACTATCCCCCGCCTCGGCCGAATGGTGCGGTCCGGGAGTCTCTGGGGCGACTATTGAATGCTGACCATGGTAACAGTGATATCC : 2026
aor8g00868  : ------------------------------------------------------------------------------------------------------------------------ :    -
aor8g00878  : ------------------------------------------------------------------------------------------------------------------------ :    -

                     *      2060         *      2080         *      2100         *      2120         *      2140
AF131g00222 : GCCAGTGTATGGCGAAGATGTGGGCGTATGAACAGGATATGAAAGCATGGACAACCAAGGCCGGAGAAACAATCAACAAGCTTTGCAAACGCCAGGGGAACTAA : 2139
AF131g00232 : GCCAGTGTATGGCGAAGATGTGGGCGTATGAACAGGATATGAAAGCATGGACAACCAAGGCCGGAGAAACAATCAACAAGCTTTGCAAACGCCAGGGGAACTGA : 2130
aor8g00868  : -------------------------------------------------------------------------------------------------------- :    -
aor8g00878  : -------------------------------------------------------------------------------------------------------- :    -
```

b

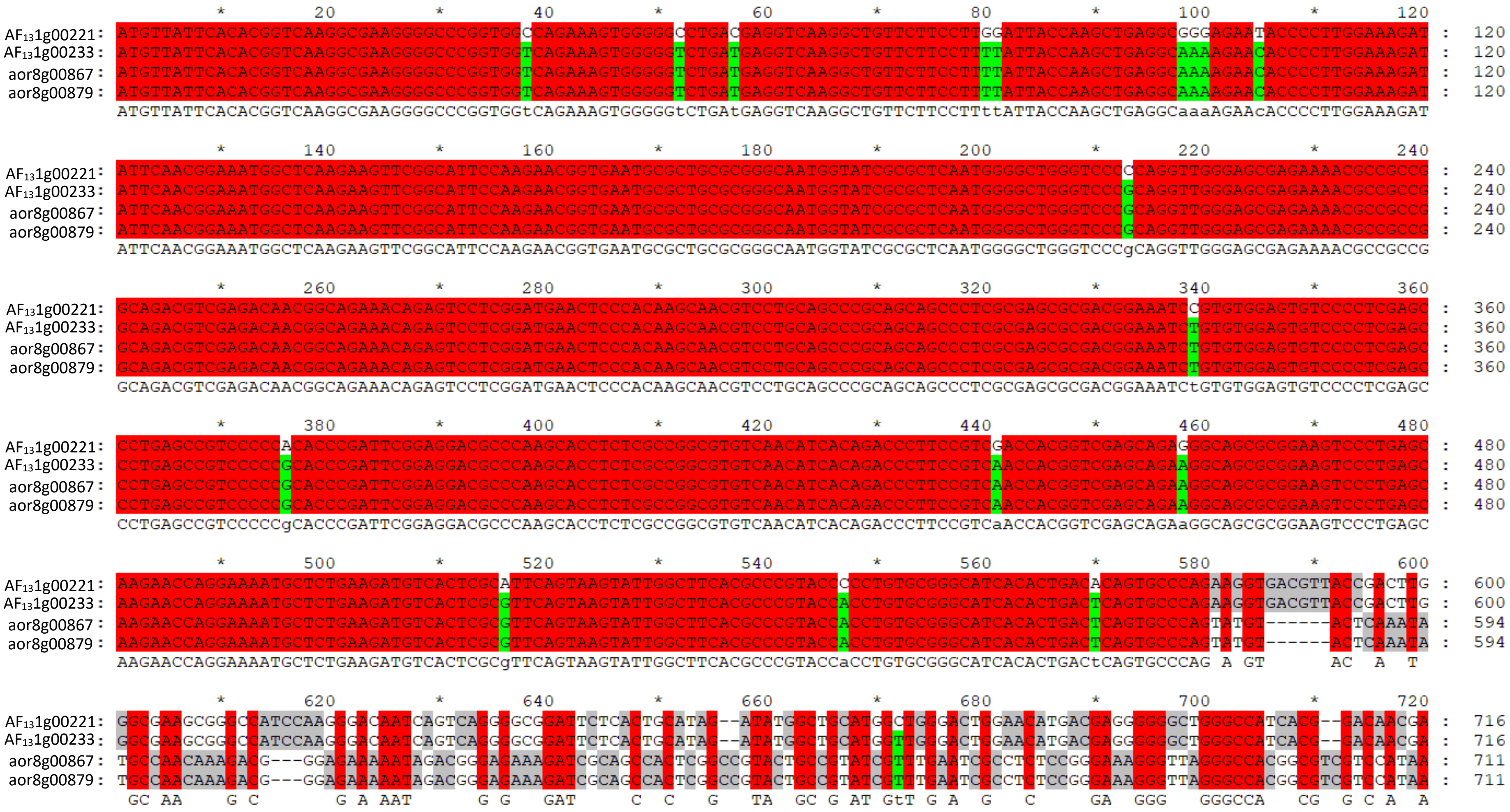

* 620 * 640 * 660 * 680 * 700 * 720

AF_{13}1g00221: GGCGAAGCGGGCCATCCAAGGGACAATCAGTCAGGGGCGGATTCTCACTGCATAGATATGGCTGCATGGCTGGGACTGGAACATGACGAGGGGGGCTGGGCCATCACGGACAACGACGCG : 720
AF_{13}1g00233: GGCGAAGCGGGCCATCCAAGGGACAATCAGTCAGGGGCGGATTCTCACTGCATAGATATGGCTGCATGGTTGGGACTGGAACATGACGAGGGGGGCTGGGCCATCACGGACAACGACGCG : 720
aor8g00866: ---ATGGCTGCATGGTTGGGACTGGAACATGACGAGGGGGGCTGGGCCATCACGGACAACGACGCG : 63
aor8g00880: ---ATGGCTGCATGGTTGGGACTGGAACATGACGAGGGGGGCTGGGCCATCACGGACAACGACGCG : 63
ATGGCTGCATGGtTGGGACTGGAACATGACGAGGGGGGCTGGGCCATCACGGACAACGACGCG

* 740 * 760 * 780 * 800 * 820 * 840

AF_{13}1g00221: ATGCGGAGGGTCATCAACGGAATGGACTGCCCTGCCAATCAGCACCCTTCCATCGCGCTCTTTGTAGGACACACCGCCAAGGCGCAGGCGCTCCGATCGCTGTATCCGCATAACAATACT : 840
AF_{13}1g00233: ATGCGGAGGGTCATCAACGGAATGGACTGCCCTGCCAATCAGCACCCTTCCATCGCGCTCTTTGTAGGACACACCGCCAAGGCGCAGGCGCTCCGATCGCTGTATCCGCATAACAATACT : 840
aor8g00866: ATGCGGAGGGTCATCAACGGAATGGACTGCCCTGCCAATCAGCACCCTTCCATCGCGCTCTTTGTAGGACACACCGCCAAGGCGCAGGCGCTCCGATCGCTGTATCCGCATAACAATACT : 183
aor8g00880: ATGCGGAGGGTCATCAACGGAATGGACTGCCCTGCCAATCAGCACCCTTCCATCGCGCTCTTTGTAGGACACACCGCCAAGGCGCAGGCGCTCCGATCGCTGTATCCGCATAACAATACT : 183
ATGCGGAGGGTCATCAACGGAATGGACTGCCCTGCCAATCAGCACCCTTCCATCGCGCTCTTTGTAGGACACACCGCCAAGGCGCAGGCGCTCCGATCGCTGTATCCGCATAACAATACT

* 860 * 880 * 900 * 920 * 940 * 960

AF_{13}1g00221: GGCCGTCACCGGAAGCAGGGTTGGGCTCAACTGCACCTGTCCGGCGCGGCCACATCTCATCCGGTTATAGTCATCGAGAGCAGCCTCTCGCGCACGTCCCCGCCCGGGCCATCCCAGCAG : 960
AF_{13}1g00233: GGCCGTCACCGGAAGCAGGGTTGGGCTCAACTGCACCTGTCCGGCGCGGCCACATCTCATCCGGTTATAGTCATCGAGAGCAGCCTCTCGCGCACGTCCCCGCCCGGGCCATCCCAGCAG : 960
aor8g00866: GGCCGTCACCGGAAGCAGGGTTGGGCTCAACTGCACCTGTCCGGCGCGGCCACATCTCATCCGGTTATAGTCATCGAGAGCAGCCTCTCGCGCACGTCCCCGCCCGGGCCATCCCAGCAG : 303
aor8g00880: GGCCGTCACCGGAAGCAGGGTTGGGCTCAACTGCACCTGTCCGGCGCGGCCACATCTCATCCGGTTATAGTCATCGAGAGCAGCCTCTCGCGCACGTCCCCGCCCGGGCCATCCCAGCAG : 303
GGCCGTCACCGGAAGCAGGGTTGGGCTCAACTGCACCTGTCCGGCGCGGCCACATCTCATCCGGTTATAGTCATCGAGAGCAGCCTCTCGCGCACGTCCCCGCCCGGGCCATCCCAGCAG

* 980 * 1000 * 1020 * 1040 * 1060 * 1080

AF_{13}1g00221: GAGCCACTCCGGCGATACCCCATCCCCCGCACTCGGGGACGCTCCTACGAGGATCTTCAGTCACTGCTGTACCGAGACGTCCTACTCCCGATCGTGGACACCGTGTGCGTCTTCGCAGCC : 1080
AF_{13}1g00233: GAGCCACTCCGGCGATACCCCATCCCCCGCACTCGGGGACGCTCCTACGAGGATCTTCAGTCACTGCTGTACCGAGACGTCCTACTCCCGATCGTGGACACCGTGTGCGTCTTCGCAGCC : 1080
aor8g00866: GAGCCACTCCGGCGATACCCCATCCCCCGCACTCGGGGACGCTCCTACGAGGATCTTCAGTCACTGCTGTACCGAGACGTCCTACTCCCGATCGTGGACACCGTGTGCGTCTTCGCAGCC : 423
aor8g00880: GAGCCACTCCGGCGATACCCCATCCCCCGCACTCGGGGACGCTCCTACGAGGATCTTCAGTCACTGCTGTACCGAGACGTCCTACTCCCGATCGTGGACACCGTGTGCGTCTTCGCAGCC : 423
GAGCCACTCCGGCGATACCCCATCCCCCGCACTCGGGGACGCTCCTACGAGGATCTTCAGTCACTGCTGTACCGAGACGTCCTACTCCCGATCGTGGACACCGTGTGCGTCTTCGCAGCC

* 1100 * 1120 * 1140 * 1160 * 1180 * 1200

AF_{13}1g00221: GACTGCGGCGGCATCCGTCACGTCCAACACTTGCTCGCATCGTGGGGCTCGCTACCGCCGACCGGTCTGGACGGCGCAGCGGCTTCCGTGCGTCCCCGTTGGGTGATTGTCCTGACAGAC : 1200
AF_{13}1g00233: GACTGCGGCGGCATCCGTCACGTCCAACACTTGCTCGCATCGTGGGGCTCGCTACCGCCGACCGGTCTGGACGGCGCAGCGGCTTCCGTGCGTCCCCGTTGGGTGATTGTCCTGACAGAC : 1200
aor8g00866: GACTGCGGCGGCATCCGTCACGTCCAACACTTGCTCGCATCGTGGGGCTCGCTACCGCCGACCGGTCTGGACGGCGCAGCGGCTTCCGTGCGTCCCCGTTGGGTGATTGTCCTGACAGAC : 543
aor8g00880: GACTGCGGCGGCATCCGTCACGTCCAACACTTGCTCGCATCGTGGGGCTCGCTACCGCCGACCGGTCTGGACGGCGCAGCGGCTTCCGTGCGTCCCCGTTGGGTGATTGTCCTGACAGAC : 543
GACTGCGGCGGCATCCGTCACGTCCAACACTTGCTCGCATCGTGGGGCTCGCTACCGCCGACCGGTCTGGACGGCGCAGCGGCTTCCGTGCGTCCCCGTTGGGTGATTGTCCTGACAGAC

* 1220 * 1240 * 1260 * 1280 * 1300 * 1320

AF_{13}1g00221: CCCGACGATGATGCAGTCCCGGCGGGAGGGATAGAAACCACGCTGCAGGCCACGGCTGTGCCCCATCTCGCGGGATCCGTCGCGGTTGTCGACCTGCGGCCCCGGCAGCCGTTGTCGCGG : 1320
AF_{13}1g00233: CCCGACGATGATGCAGTCCCGGCGGGAGGGATAGAAACCACGCTGCAGGCCACGGCTGTGCCCCATCTCGCGGGATCCGTCGCGGTTGTCGACCTGCGGCCCCGGCAGCCGTTGTCGCGG : 1320
aor8g00866: CCCGACGATGATGCAGTCCCGGCGGGAGGGATAGAAACCACGCTGCAGGCCACGGCTGTGCCCCATCTCGCGGGATCCGTCGCGGTTGTCGACCTGCGGCCCCGGCAGCCGTTGTCGCGG : 663
aor8g00880: CCCGACGATGATGCAGTCCCGGCGGGAGGGATAGAAACCACGCTGCAGGCCACGGCTGTGCCCCATCTCGCGGGATCCGTCGCGGTTGTCGACCTGCGGCCCCGGCAGCCGTTGTCGCGG : 663
CCCGACGATGATGCAGTCCCGGCGGGAGGGATAGAAACCACGCTGCAGGCCACGGCTGTGCCCCATCTCGCGGGATCCGTCGCGGTTGTCGACCTGCGGCCCCGGCAGCCGTTGTCGCGG

* 1340 * 1360 * 1380 * 1400 * 1420 * 1440

AF_{13}1g00221: TTGAGCCGGTTCGAGCCTCTCCGGCGACGGCTGTCACTGGAGCTGGACGAGGTGCGCACGCTGCGTGCCAACGCACATCTCCTCTTCTCCGCCCTCCATCTGGAGTGGATCTTCCGGGGA : 1440
AF_{13}1g00233: TTGAGCCGGTTCGAGCCTCTCCGGCGACGGCTGTCACTGGAGCTGGACGAGGTGCGCACGCTGCGTGCCAACGCACATCTCCTCTTCTCCGCCCTCCATCTGGAGTGGATCTTCCGGGGA : 1440
aor8g00866: TTGAGCCGGTTCGAGCCCCTCCGGCGACGGCTGTCACTGGAGCTGGACGAGGTGCGCACGCTGCGTGCCAACGCACATCTCCTCTTCTCCGCCCTCCATCTGGAGTGGATCTTCCGGGGA : 783
aor8g00880: TTGAGCCGGTTCGAGCCCCTCCGGCGACGGCTGTCACTGGAGCTGGACGAGGTGCGCACGCTGCGTGCCAACGCACATCTCCTCTTCTCCGCCCTCCATCTGGAGTGGATCTTCCGGGGA : 783
TTGAGCCGGTTCGAGCC CTCCGGCGACGGCTGTCACTGGAGCTGGACGAGGTGCGCACGCTGCGTGCCAACGCACATCTCCTCTTCTCCGCCCTCCATCTGGAGTGGATCTTCCGGGGA

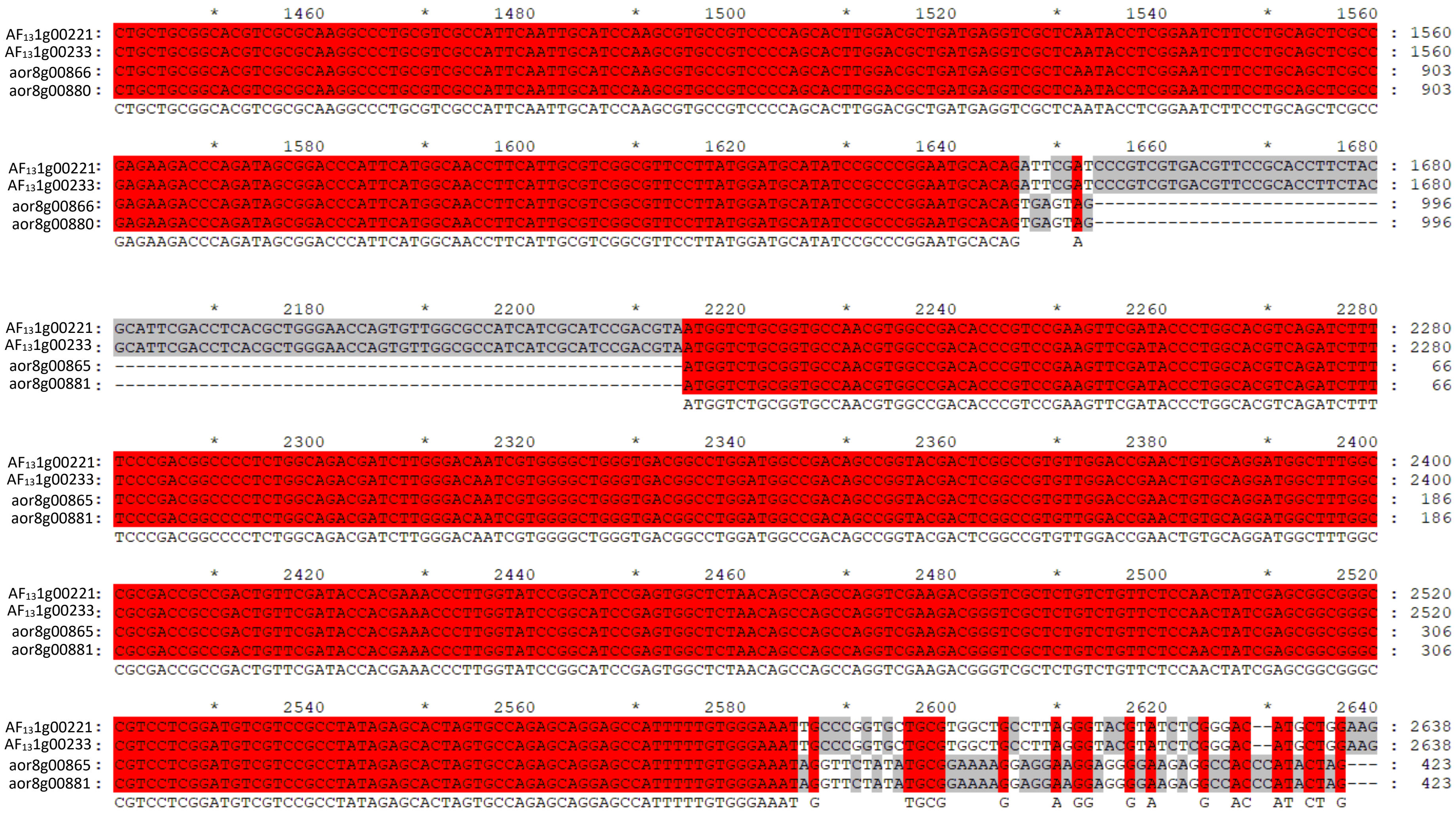

1460
1480
1500
1520
1540
1560
AF131g00221 : 1560
AF131g00233 : 1560
aor8g00866 : 903
aor8g00880 : 903
1580
1600
1620
1640
1660
1680
AF131g00221 : 1680
AF131g00233 : 1680
aor8g00866 : 996
aor8g00880 : 996
2180
2200
2220
2240
2260
2280
AF131g00221 : 2280
AF131g00233 : 2280
aor8g00865 : 66
aor8g00881 : 66
2300
2320
2340
2360
2380
2400
AF131g00221 : 2400
AF131g00233 : 2400
aor8g00865 : 186
aor8g00881 : 186
2420
2440
2460
2480
2500
2520
AF131g00221 : 2520
AF131g00233 : 2520
aor8g00865 : 306
aor8g00881 : 306
2540
2560
2580
2600
2620
2640
AF131g00221 : 2638
AF131g00233 : 2638
aor8g00865 : 423
aor8g00881 : 423

C

	* 20 * 40 * 60 * 80 * 100 * 120	
AF$_{13}$1g00240 :	ATGGAACACGATACGCAGGACTCCCCAGAGCCGCGAGTAACCCCCAATCAGCCCATCCACGCTGGCGTGGAGGGACCGACCGCTCCGGAGTTGACGGCGCCCCGCAGCATTGCCCTGGGC :	120
AF$_{13}$1g00218 :	ATGGAATACGACACGCTGGACTCCCCAGAGCCGCGAGCAACCCCCAATCAGCCCATCCACGCTGGCGTGGAGGGACTGACCGCTCCGGAGTTGACGGCGCCCCGCAGCATTGCCCTGGGC :	120
aor8g00859 :	-- :	-
aor8g00860 :	-- :	-
aor8g00861 :	-- :	-

	* 140 * 160 * 180 * 200 * 220 * 240	
AF$_{13}$1g00240:	TTGACCCGTGACTATGCGGCCAACTGGACGGTTCCCGATGCCCTGCGGGAGCTCTATCAAAACTGGAAGGACGCTATCCTCCAGACCCACTCGATCAGTCTGCTCGAGTTTGCACCCCGG :	240
AF$_{13}$1g00218:	TTGACCCGTGACTATGCGGCCAACTGGACGGTTCCCGATGCCCTGCGGGAGCTCTATCAAAACTGGAAGGACGCCATCCTCCAGACCCACTCGATCAGCCTGCTCGAGTTTGCCCCCCGG :	240
aor8g00859 :	-- :	-
aor8g00860 :	-- :	-
aor8g00861 :	-- :	-

	* 260 * 280 * 300 * 320 * 340 * 360	
AF$_{13}$1g00240:	GTGTCGGCAACGTCGGACGCCATCACGATTGTCGTGGAGGGGTTGCAACCATCCTTCGGCGGGGGCGGCGGAGGGCCCCTGGTCCGAAGGATCTTGGGATATATTCGCTTCAACAAGCCC :	360
AF$_{13}$1g00218:	GTGACGGCAACGTCGGACGCCATCACGATTGTCGTGGAGGGGTTGCAGCCACCCTTCGGCGGGGGCGGCGGAGGGCCCCTGGTCCGGAGGATCTTGGGATATATTCGCTTCAACAAGCCT :	360
aor8g00859 :	-- :	-
aor8g00860 :	-- :	-
aor8g00861 :	-- :	-

	* 380 * 400 * 420 * 440 * 460 * 480	
AF$_{13}$1g00240 :	CAAGGCAGTGCGGAGTTCACCAATTTTAGATCCTCTCTGGACCCGCAATGCTTGGCGATCGGTCACAGCAGCAAGAAGCAAGACGAGCGCCTGGCCGGTGGTCACGGAGAGGGGCTGAAG :	480
AF$_{13}$1g00218 :	CAAGGCAGTGCGGAGTTCACCAATTTTAGATCCTCTCTGGACCCGCAATGCTTGGCGATCGGTCACAGCAGCAAGAAGCAAGACGAGCGCCTGGCCGGTGGTCACGGAGAGGGGCTGAAG :	480
aor8g00859 :	-- :	-
aor8g00860 :	-- :	-
aor8g00861 :	-- :	-

	* 500 * 520 * 540 * 560 * 580 * 600	
AF$_{13}$1g00240 :	ATCGCCGCGTTGGTCCTCTCGCGGGCGGAGCATCACGTCAAGATCTCCGCGAGCGGGACCTACTGGAACTTTGGCTTCAACGGCCACTCGAAATCGAACTTTTACTGCCGGCTTTCACCG :	600
AF$_{13}$1g00218 :	ATCGCCGCGTTGGTCCTCTCGCGGGCGGAGCATCACGTCAAGATCTCCGCGAGCGGGACCTACTGGAACTTTGGCTTCAACGGCCACTCGAAATCGAACTTTTACTGCCGGCTGTCACCG :	600
aor8g00859 :	-- :	-
aor8g00860 :	-- :	-
aor8g00861 :	-- :	-

	* 620 * 640 * 660 * 680 * 700 * 720	
AF$_{13}$1g00240 :	GCCAAGGCGAAGCGGGATCCCGGGCCCGCCAGCGACGGCATTCTGCTCCCCCGGCTGACCGCCGAGGTCGGACGGGACGTCTCGGTGCTGGTCGAGAAAGGCCAGAAGGGCCAGCGGTTG :	720
AF$_{13}$1g00218 :	GCCAAGGCGAAGCGGGATCCCGGGCTCGCCAGCGACGGCATTCTGCTCCCCCGGCTGACCGCCGAGGTCGGACGGGACGTCTCGGTGCTGGTCGAGAAAGGCCAGAAGGGCCAGCGGTTG :	720
aor8g00859 :	-- :	-
aor8g00860 :	-- :	-
aor8g00861 :	-- :	-

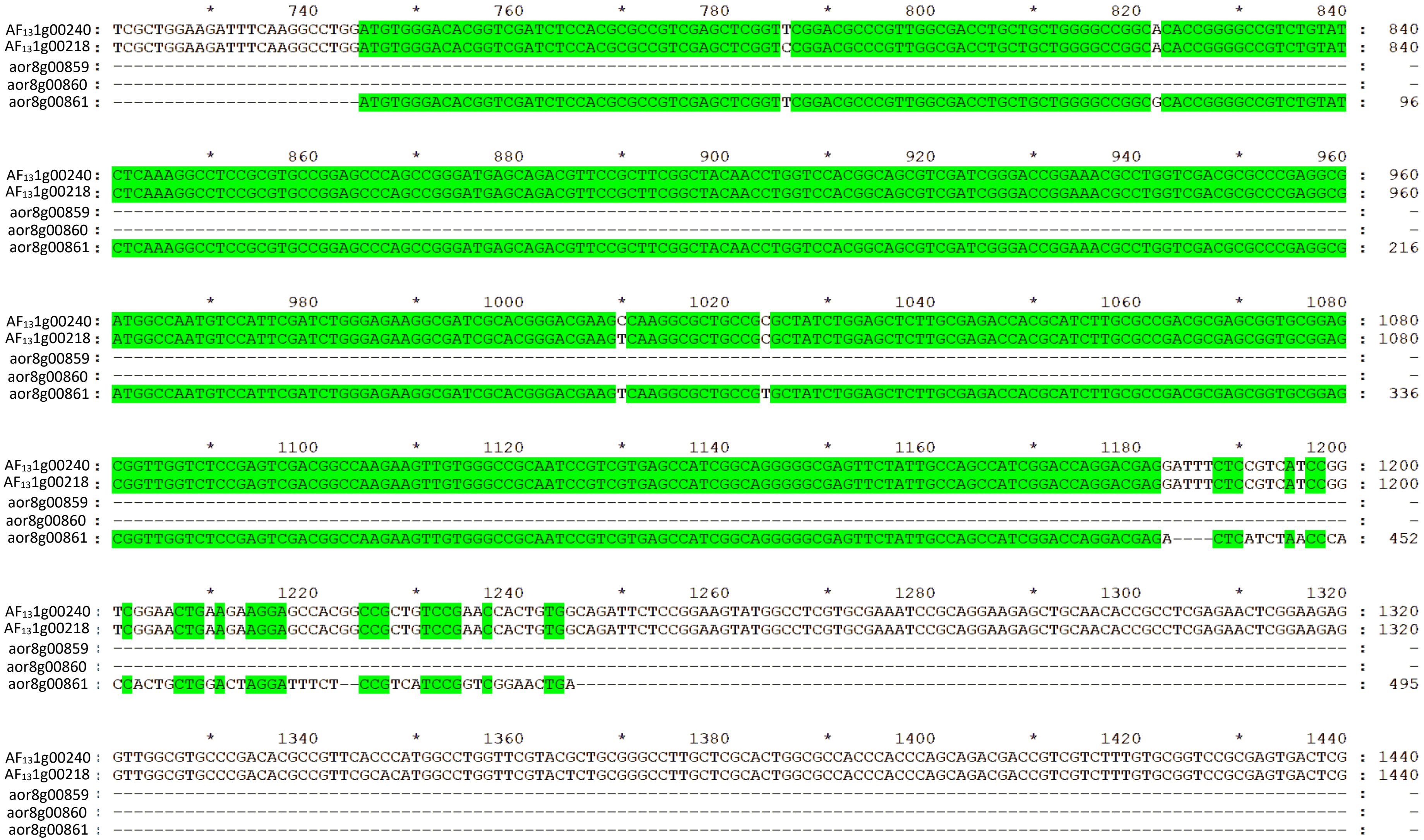
AF131g00240
AF131g00218
aor8g00859
aor8g00860
aor8g00861

```
                 *       1460        *       1480        *       1500        *       1520        *       1540        *       1560
AF13 1g00240 : GTCGATATGGCCTATCGCATGGAAGCCAACGCACTCTACATCCATGAGAAGTGGCTTCATGTTCGGGGCGCCGAAGGCCCGGCAACGGAAGGCGCCATCCGCTCGGAGCGCGACGTGTTT : 1560
AF13 1g00218 : GTTGATATGGCCTATCGCATGGAAGCCAACGCACTCTACATCCATGAGAAGTGGCTTCATGTTCGGGGCGCCGATGGCCCGGCAACGGAAGGCGCCATCTGCTCGGAGCGCGACGTGTTT : 1560
aor8g00859 : ------------------------------------------------------------------------------------------------------------------------ :    -
aor8g00860 : ------ATGGCCTATCGCATGGAAGCCAACGCACTCTACATCCATGAGAAGTGGCTTCATGTTCGGGGCGCCGAAGGCCCGGCAACGGAAGGCGCCATCCGCTCGGAGCGCGACGTGTTT :  114
aor8g00861 : ------------------------------------------------------------------------------------------------------------------------ :    -

                 *       1580        *       1600        *       1620        *       1640        *       1660        *       1680
AF13 1g00240 : CTCTGCCAGCATCTGGCGGAAGAGCTGTACCGGCGTGCCGTGACGCTCATCGTCCACAAGACCGACGGGCCGCGCACGAGCCAGACAATCCAGCCGCTCCTTCAAGTGGCACACTGGAAG : 1680
AF13 1g00218 : CTCTGCCAGTATCTGGCGGAAGAGCTGTACCGGCGTGCCGTGACGCTCATCGTCCACAAGACCGACGGGCCGCGCACGAGCCAGACAATCCAGCCGCTCCTTCAAGTGGCACACTGGAAG : 1680
aor8g00859 : ------------------------------------------------------------------------------------------------------------------------ :    -
aor8g00860 : CTCTGCCAGCATCTGGCGGAAGAGCTGTACCGGCGTGCCGTGACGCTCATCGTCCACAAGACCGACGGGCCGCGCACGAGCCAGACAATCCAGCCGCTCCTTCAAGTGGCACACTGGAAG :  234
aor8g00861 : ------------------------------------------------------------------------------------------------------------------------ :    -

                 *       1700        *       1720        *       1740        *       1760        *       1780        *       1800
AF13 1g00240 : TTGCACGAGATGCCACGCCAGATTGAGGTGTACGCCGCCGCCGCTGACGACGGCCCGAGGACGACACAGGTCTCGTTCTACACGGGCCACAGCCTCCTGTTCACTAAGCTGTGCGGGACG : 1800
AF13 1g00218 : TTGCACGAGATGCCACGCCGGATTGAGGTGTACGCCGCCGCCGCTGACGACGGACCGAGGGCGACACAGGTCTCGTTCTACACGGGCCACAGCCTCCTGTTCACTAAGCTATGCGGGACG : 1800
aor8g00859 : ------------------------------------------------------------------------------------------------------------------------ :    -
aor8g00860 : TTGCACGAGATGCCACGCCGGATTGAGGTGTACGCCGCCGCCGCTGACGACGGACCGAGGGCGACACAGGTCTCGTTCTACACGGGCCACAGCCTCCTGTTCACTAAGCTATGCGGGACG :  354
aor8g00861 : ------------------------------------------------------------------------------------------------------------------------ :    -

                 *       1820        *       1840        *       1860        *       1880        *       1900        *       1920
AF13 1g00240 : CAAGTGTCGTATCTGGTCGTGCTACATGGGCCCAACTGCGTCGCGCAGTCGGTGGATCACCTGCTGTACGACCCCAGCCGGGACTGCTGTCTGTGCCCGCGGCAGGTGGTGCCCCTGAGC : 1920
AF13 1g00218 : CAAGTGTCGTATCTGGTCGTGCTACATGGGCCCAACTGCGTCGCGCAGTCGGTGGATCACCTGCTGTACGACCCCAGCCGGGACTGCTGTCTGTGCCCGCGGCAGGTGGTGCCCCTGAGC : 1920
aor8g00859 : ------------------------------------------------------------------------------------------------------------------------ :    -
aor8g00860 : CAAGTGTCGTATCTGGTCGTGCTACATGGGCCCAACTGCGTCGCGCAGTCGGTGGATCACCTGCTGTACGACCCCAGTCGGGACTGCTGTCTGTGCCCGCGGCAGGTGGTGCCCCTGAGC :  474
aor8g00861 : ------------------------------------------------------------------------------------------------------------------------ :    -

                 *       1940        *       1960        *       1980        *       2000        *       2020        *       2040
AF13 1g00240 : ATGCGACGAGCTGTGTTCGAGGATCCTGGCCACGGGCCCTGGGTTCCCATGGTGGTGAAAATGTCGGTTCGTGACGGCCTTGTCGGCGCCATGTCCTCCCCAGGGGGCCATCATTGGCCG : 2040
AF13 1g00218 : ATGCGACGAGCTGTGTTCGAGGATCCTGGCCACGGGCCCTGGGTTCCCATGGTGGTGAAGATGTCGGTTCGTGACGGCCTTGTCGGCGCCATGTCCTCCCCAGGGGGCCATCATTGGCCG : 2040
aor8g00859 : ------------------------------------------------------------------------------------------------------------------------ :    -
aor8g00860 : ATGCGACGAGCTGTGTTCGAGGATCCTGGCCACGGGCCCTGGGTTCCCATGGTGGTGAAGATGTCGGTTCGTGACGGCCTTGTCGGCGCCATGTCCTCCCCAGGGGGCCATCATTGGCCG :  594
aor8g00861 : ------------------------------------------------------------------------------------------------------------------------ :    -

                 *       2060        *       2080        *       2100        *       2120        *       2140        *       2160
AF13 1g00240 : CTTCTTCCGAAAGCGCAGGACCGAGCGTTGGTGGCGATTGCGCCTCCTTCCCACTCTCCGTCATCGGAGACTGCAGCTCCCCGCGGGACCGTCCCGGTGGATCGTCCGCCCACGGTCAGT : 2160
AF13 1g00218 : CTTCTTCCGAAAGCGCAGGACGGAGCGTTGGTGGCGATTGCGCCTCCTTCCCACTCTTCGTCATCGGAGACTGCAGCTCCCCGCGGGACCGTCCCGGTGGATCGTCCGCCCACGGTCTGT : 2160
aor8g00859 : ------------------------------------------------------------------------------------------------------------------------ :    -
aor8g00860 : CTTCTTCCGAAAGCGCAGGACGGAGCGTTGGTGGCGATTGCGCCTCCTTCCCACTCTCCGTCATCGGAGACTGCAGCTCCCCGCGGGACCGTCCCGGTGGATCGTCCGCCCACGGTCTGT :  714
aor8g00861 : ------------------------------------------------------------------------------------------------------------------------ :    -
```

```
                        *      2180         *      2200         *      2220         *      2240         *      2260         *      2280
AF131g00240 : GCTGCCTTGGATCCTGTGCTCACCACTCCGACGGCTGCCAGCCAGGAGAACCAAGAAACGACAGCCGCGTCGCAGCTGGCCGCTAGGCCGCTTGAACTGGAAACGGGCGCGCAGACGACG : 2280
AF131g00218 : GCTGCCTTGGATCCTGTGCTCACCACCCCGACGGCTGCCAGCCAGGAGAACCAAGAAACGACAGCCGCGTCGCAGCTGGCCGCTAGGCCACTTGAACTGGAAACGGGCGCGCAGACGACG : 2280
aor8g00859  : ------------------------------------------------------------------------------------------------------------------------ :    -
aor8g00860  : GCTGCCTTGGATCCTGTGCTCACCACCCCGACGGCTGCCAGCCAGGAGAACCAAGAAACGACAGCCGCGTCGCAGCTGGCCGCTAGGCCGCTTGAACTGGAAACGGGCGCGCAGACGACG :  834
aor8g00861  : ------------------------------------------------------------------------------------------------------------------------ :    -

                        *      2300         *      2320         *      2340         *      2360         *      2380         *      2400
AF131g00240 : GCATCGGACACCGCATCAGACACCGCATCAGACACCGCA------------GCACCGGTCCCTCTCGTATCAGTGGTGGAATGCCTCGCCCCCCACCCGGATGACCATCCCGAGTCCGCG : 2388
AF131g00218 : GCATCGGACACCGCATCAGACACCGCATCAGACACCGCATCAGACACCACAGCGCCGGTCCCTCTCGTATCAGTGGTGGAATGCCTCGCCCC------GGATGACCATCCTGAGTCCGCG : 2394
aor8g00859  : ------------------------------------------------------------------------------------------------------------------------ :    -
aor8g00860  : GCATCGGACACCGCATCAGACACCGCATCGGACACCGCA------------GCACCGGTCCCTCTCGTACCAGTGGTGGAAAGCCTCGCCCCCCACCCGGATGACCATCCCGAGTCCGCG :  942
aor8g00861  : ------------------------------------------------------------------------------------------------------------------------ :    -

                        *      2420         *      2440         *      2460         *      2480         *      2500         *      2520
AF131g00240 : TCTGGTGCGCTGCCCTCGACCATCTCCCGGAATATGACCTCTGCGGATGGTGCGTGGTGGCAGACATGGCCAGAACCGCCCCGGGATGTCCAAACGTCACCGAGCGTTGCGGATCACAAC : 2508
AF131g00218 : TCAAATGCGCTGCCCTCGACCATCTCCGGGAATATGACCTCTGCGGATGGTGCGTGGTGGCAGACCTGGCCAGAACCACCCCGGGATGTCCAAACGTCACCGAGCGCTGCGGATCAGAAC : 2514
aor8g00859  : ------------------------------------------------------------------------------------------------------------------------ :    -
aor8g00860  : TCTGGTGCGCTGCCCTCGACCATCTCCCGGAATATGACCTCTGCGGATCGTGCGTGGTGGCAGACCTGGCCAGAACCGCCCCGGGATGTCCAAACGTCACCAAGCGCTGCGGATCACAAC : 1062
aor8g00861  : ------------------------------------------------------------------------------------------------------------------------ :    -

                        *      2540         *      2560         *      2580         *      2600         *      2620         *      2640
AF131g00240 : ATTGGACGCTTTCA---------------------------------------------------------------------------------------------------------G : 2523
AF131g00218 : ATTGGACGCTTTCATTCTAGCTGGGTCTCCTCCCTCTCGCCGGATCTGCTCCGCCGTTTCCCTACAAACCCCTTGCTCTCCCGTTGCACGAGGGGACGCCGTGGTTTAACAATCCTGCAG : 2634
aor8g00859  : ------------------------------------------------------------------------------------------------------------------------ :    -
aor8g00860  : ATTGGACGCTTTCAG-----------------------------------------------------------------------------------------------------GTAA : 1081
aor8g00861  : ------------------------------------------------------------------------------------------------------------------------ :    -

                        *      2660         *      2680         *      2700         *      2720         *      2740         *      2760
AF131g00240 : GTCCAGGAATCGCATCGGTCATGCGAGCCACCCCAGGGCCTCTCGAAAGGGCAATACGCTCGGGTCCAGCTCCGCGAGCCGGGCGGGGATGGCACCACGACCGGTCGGTACGTCCTCTAC : 2643
AF131g00218 : GTCCAGGAATCGCATCGGTCATGCGAGCCACCCCAGGGCCTCTCGAAAGGGCAATACGCTCGGGTCCAGCTCCGCGAGCCAGGCGGGGATAGCACCACGACCGGTCGGTACGTCCTCTAC : 2754
aor8g00859  : ------------------------------------------------------------------------------------------------------------------------ :    -
aor8g00860  : GTTCTCCCTTGTCAT--ATCATGTCCTTCACCACCGGTGCCCTTCTTCCAATATCATCCTT--CCCTGTCCTTTCGATAAAGTCTAGAA------------------------------- : 1166
aor8g00861  : ------------------------------------------------------------------------------------------------------------------------ :    -

                        *      2780         *      2800         *      2820         *      2840         *      2860         *      2880
AF131g00240 : GTCCACGATGTTCTGCCGCCGACCCCGTCGCATGGTTCGGGTGCCCGTCTCGTCGTCACGAAATACTCCTTCCTGGCCGACCGGTTAGCGTGGGAAGCCGGGCATCCAGACACTCAGGTC : 2763
AF131g00218 : GTCCACGATATTCTGCCGCCGACACCGTCGCATGGTCCGGGTGCCCGTCTCGTCGTCACGAAATACTCCTTCCTGGCCGACCGGTTAGCATGGGAAGCCGGGCATCCAGACACTCAGGCC : 2874
aor8g00859  : ------------------------------------------------------------------------------------------------------------------------ :    -
aor8g00860  : ------------------------------------------------------------------------------------------------------------------------ :    -
aor8g00861  : ------------------------------------------------------------------------------------------------------------------------ :    -
```

```
                      *      2900         *      2920         *      2940         *      2960         *      2980         *      3000
AF131g00240: CAGGAGCTGCTCCTGCACTTCCGGGACGCTGCCCGGATTGGCTATCCGGACGACGCGGAGATTATTAATGTCGACACCGTGGTCGCGGTGGATTCGATGGACACCGAGCCGGTTGGCCCG : 2883
AF131g00218: CAGGAGCTGCTCCTGCACTTCCGGGACGCTGCCCGGATTGGCTTTCCGGACGACGCGGAGATTATTAATGTCGACACCGTGGTCGCGGTGGATTCGATGGACACCGAGCCGGTTGGCCCG : 2994
aor8g00859 : ------------------------------------------------------------------------------------------------------------------------ :    -
aor8g00860 : ------------------------------------------------------------------------------------------------------------------------ :    -
aor8g00861 : ------------------------------------------------------------------------------------------------------------------------ :    -

                      *      3020         *      3040         *      3060         *      3080         *      3100         *      3120
AF131g00240: ACCGTGTCGTATGTCGCACAGGTGCCTGACCCGGCAAACGGCCTTTTCGTGCGGTATGGCATTCGTGAGGGGTCCCGACGGGAGTGGTTGTTTCTGGCGCCGCTGGTGTCTTCACTCACG : 3003
AF131g00218: ACCGTGTCGTATGTCGCACAGGTGCCTGACCCGGCGAACGGCCTTTTCGTGCGGTATGGCATTTGTGAGGGATCCCAGCGGGAATGGTTGTTTCTGGCGCCGCTGGGGTCTTCGCTCACG : 3114
aor8g00859 : -----------------------------------------------------------------------------------ATGGTTGTTTCTGGCGCCGCTGGTGTCTTCGCTCACG :   37
aor8g00860 : ------------------------------------------------------------------------------------------------------------------------ :    -
aor8g00861 : ------------------------------------------------------------------------------------------------------------------------ :    -

                      *      3140         *      3160         *      3180         *      3200         *      3220         *      3240
AF131g00240: GGCCCGAGAGGCAGTGCCGCGTATCCACGCTTTTCACCGCTGCCCGTGGCCTCCGTCTTCGATCTCTCCCCCGACGACCTGCGCCTGTCGATTGGCTTCCAGAAGGCGGGATATTGCGTG : 3123
AF131g00218: GGCCCGAGAGGCAGTGCCGCGTATCCACGTTTTTCTCCGCTGCCCGTGGCCTCCGTCTTCGATCTCTCCCCCGACGACCTGCGCCTGTCGGTTGGCTTCCAGAAGGCGGGATATTGCGTG : 3234
aor8g00859 : GGCCCGAGAGGCAGTGCCGCGTATCCACGCTTTTCTCCGCTGCCCGTGGCCTCCGTCTTCGATCTCTCCCCCGACGACCTGCGCCTGTCGGTTGGCTTCCAGAAGGCGGGGTATTGCGTG :  157
aor8g00860 : ------------------------------------------------------------------------------------------------------------------------ :    -
aor8g00861 : ------------------------------------------------------------------------------------------------------------------------ :    -

                      *      3260         *      3280         *      3300         *      3320         *      3340         *      3360
AF131g00240: CAGGCCGCGGTCGGTTTTGCCGAAGACCGCCACCAGGCATGGAAG--------------------------------------------------------------------------- : 3168
AF131g00218: CAGGCCGCGGTTGGCTTTGCCGAAGACCGCCACCAGGCATGGAAG--------------------------------------------------------------------------- : 3279
aor8g00859 : CAGGCCGCGGTCGGTTTTGCCGAAGACCGCCACCAGGCATGGAAGGTGAGTGCGCCCTTTCCCGTCGGTGTCCAGATCGATGGGCCCCTGAGGCCACTGTAAAGGCGGCGCTGACCCTGT :  277
aor8g00860 : ------------------------------------------------------------------------------------------------------------------------ :    -
aor8g00861 : ------------------------------------------------------------------------------------------------------------------------ :    -

                      *      3380         *      3400         *      3420         *      3440         *      3460         *      3480
AF131g00240: -----------ACGCAACATCCCGATGCCACCGTCTACCCGGGCTGTGTTGCATCCGCCCTCGCGACGATGGACTACGACCTCTCCACGCAACCGGATGACAGAGGGCGTGATGTGCCCC : 3277
AF131g00218: -----------ACGCAACATCCCGATGCCACCGTCTACCCGGGCTGTGTTGCATCCGCCCTCGCGACGATGGACTACGACCTCTCCACGCAACCGGATGACAGAGGGCGTGATGTGCCCC : 3388
aor8g00859 : GTTGTCAACAGACGCAACATCCCGATGCCACCGTCTACCCGGGCTGTGTTGCATCCGCCCTCGCGACGATGGACTACGACCTCTCCATGCAACCGGATGACAGAGGGCGTGATGTGCCCC :  397
aor8g00860 : ------------------------------------------------------------------------------------------------------------------------ :    -
aor8g00861 : ------------------------------------------------------------------------------------------------------------------------ :    -

                      *      3500         *      3520         *      3540         *      3560         *      3580         *      3600
AF131g00240: GCATTGTGACCATTAGTGGCGAGGGCTCTTTCGCTAAATCAGCCGCCGGTTGGCTCGACCCTAGCTCCTCGGGCTTCGATGGCTTCGACGGTCTCTCCGGGGAGAGGCTGAGTCCGCTTC : 3397
AF131g00218: GCATTGTGACCATTAGTGGCGAGGGCTCTTTCGCTAAATCAGCCGCCGGTTGGCTCGACCCTAGCTCCTCGGGCTTCGATGGCTTCGACAGTCTCTCCAGGGAGACGCTGAGTCCGCTTC : 3508
aor8g00859 : GCATTGTGACCATTAGTGGCGAGGGCTCTTTCGCTAAATCAGCCGCCGGTTGGCTCGACCCTAGCTCCTCGGGCTTCGATGGCATCAACGGTCTCTCCGGGGAGACGCTGAGTCCGCTTC :  517
aor8g00860 : ------------------------------------------------------------------------------------------------------------------------ :    -
aor8g00861 : ------------------------------------------------------------------------------------------------------------------------ :    -
```

```
                     *      3620        *      3640        *      3660        *      3680        *      3700        *      3720
AF131g00240 : AACAGTGCGAGCTCGCGGCGCAGTCTCCAGGGCTGAGTCCGGACTTCATCGTGCTGACGATGTCCCGGTCGATCTTGACAGACCAGCGGTGGGGCTCACTGGCTCGCACCATGGGTCTTC : 3517
AF131g00218 : AACAGTGTGAGCTCGCGGCGCAGTCTCCAGGGCTGAGTCCGGACTTCATCGTGCTGACGATGTCCCGGTCGATCTTGACAGACCCGCGGTGGGGCTCACTGGCTCGCACCATGGGTCTTC : 3628
aor8g00859  : AACAGTGTGAGCTCGCGGCGCAGTCTCCAGGGCTGAGTCCGGACTTCATCGTGCTGACGATGTCCCGGTCGATCTTGACAGACCAGTTGTGGGGCTCACTGGCTCGCACCATGGGTCTTC :  637
aor8g00860  : ------------------------------------------------------------------------------------------------------------------------ :    -
aor8g00861  : ------------------------------------------------------------------------------------------------------------------------ :    -

                     *      3740        *      3760        *      3780        *      3800        *      3820        *      3840
AF131g00240 : TCCTCGAGCAAGGGTATGCGGTGGCGCTGCGGCCGGTCTCTCTGCCGGATCCGGGGCAGCATGGGGAGCGATCGGTTCTCCTCCTCGCCGCCCCGGGCCGCACCGACCCACAGTGGATCG : 3637
AF131g00218 : TCCTCGAGCAAGGGTATGCGGTGGCGCTGCGGCCGGTCTCTCTGCCGGATCCGGGGCAGCATGGAGAGCGATCGGTTCTCCTCCTCGCCGCCCCGGGCCGCACCGACCCACAGTGGATCG : 3748
aor8g00859  : TCCTCGAGCAAGGGTATGCGGTGGCGCTGCGGCCGGTCTCTCTGCCGGATCCGGGGCAGCGTGGCGAGCGATCGGTTCTCCTCCTCGCCGCCCCGGGCCGCACCGACCCACAGTGGATCG :  757
aor8g00860  : ------------------------------------------------------------------------------------------------------------------------ :    -
aor8g00861  : ------------------------------------------------------------------------------------------------------------------------ :    -

                     *      3860        *      3880        *      3900        *      3920        *      3940        *      3960
AF131g00240 : ATGACACGCTTTCCGACCTGCGAATGGTATCAGCCTCGGACGAGCTGCTGGAACGTGCGGAGAACGAGAGCGGCGTCTCTAGCGTCCCGGCCGCGGATGGCATAGCGGCAGCCGGGTCGG : 3757
AF131g00218 : ATGACACGCTTTCCGACCTGCGAATGGTATCAGCCTCGGACGAGCTGCTGGAACGTGCGGAGAACGAGAGCGGCGTCTCTAGCGTCCCGGCCGCGGATGGCATAGCGGCAGCCGGGTCGG : 3868
aor8g00859  : ATGACACGCTTTCCGACCTGCGAATGGTGTCGGCCTCGGACGAGCTGCTGGAACGTGCGGAGAACGAGAGCGGCGTCTCTAGCGTCCCGGCCGTGGACGATGTAGCGGCAGCCGAGTCGG :  877
aor8g00860  : ------------------------------------------------------------------------------------------------------------------------ :    -
aor8g00861  : ------------------------------------------------------------------------------------------------------------------------ :    -

                     *      3980        *      4000        *      4020        *      4040        *      4060        *      4080
AF131g00240 : CGGATGTAGGCGATGCTAGTCCTCCAGCGAATTCTACTCCTCAGCATGGTGACAGCGTTCTCGCGGATGGGGCACAACATATTGCCGCCACGGTCTCCCGGATTATCGGAGAATTTTCAA : 3877
AF131g00218 : CGGATGTAGGCGTTGCTAGTCCTCCAGCGAATTCTACTCCTCGGCATGGTGACAGCGTTCTCGCGGATGGCGCACAACATATTACCGCCACGGTCTCCCGGATTATTGGAGAATTTTCAA : 3988
aor8g00859  : CGGGTGGAGGCGATGCTAGTCCTCCATCGGATTCTACTCCTCGGCGTGGTGACAGCGTTCGCGCGGATGGCGCACAACATATTGCCGCCACGGTCTCCCGGATTATCGGAGAATTCTCAA :  997
aor8g00860  : ------------------------------------------------------------------------------------------------------------------------ :    -
aor8g00861  : ------------------------------------------------------------------------------------------------------------------------ :    -

                     *      4100        *      4120        *      4140        *      4160        *
AF131g00240 : CCAAGTGCGCGCTGACGACGAACCTCCCCCGGTTGGTTGTGACCGACAATTCGGATGGCGCGCAGGACAGAGCCAAGCGACAGAGGATGTAG : 3969
AF131g00218 : CCAAGTGCGCGCTGACGACGAACCTCCCCCGGTTGGTTGTGACCGACAATTCGGATGGCGCGCAGAACAGAGCCAAGCGGCAGAGGATGTAG : 4080
aor8g00859  : CCAAGTGCGCGCTGACGACGAACCTCCCCCGGTTGGTAGTGACCGACAATTCGGATGGCGCGCAGGACAGAGCCAAGCGACAGAGGATGTAG : 1089
aor8g00860  : -------------------------------------------------------------------------------------------- :    -
aor8g00861  : -------------------------------------------------------------------------------------------- :    -
```

d

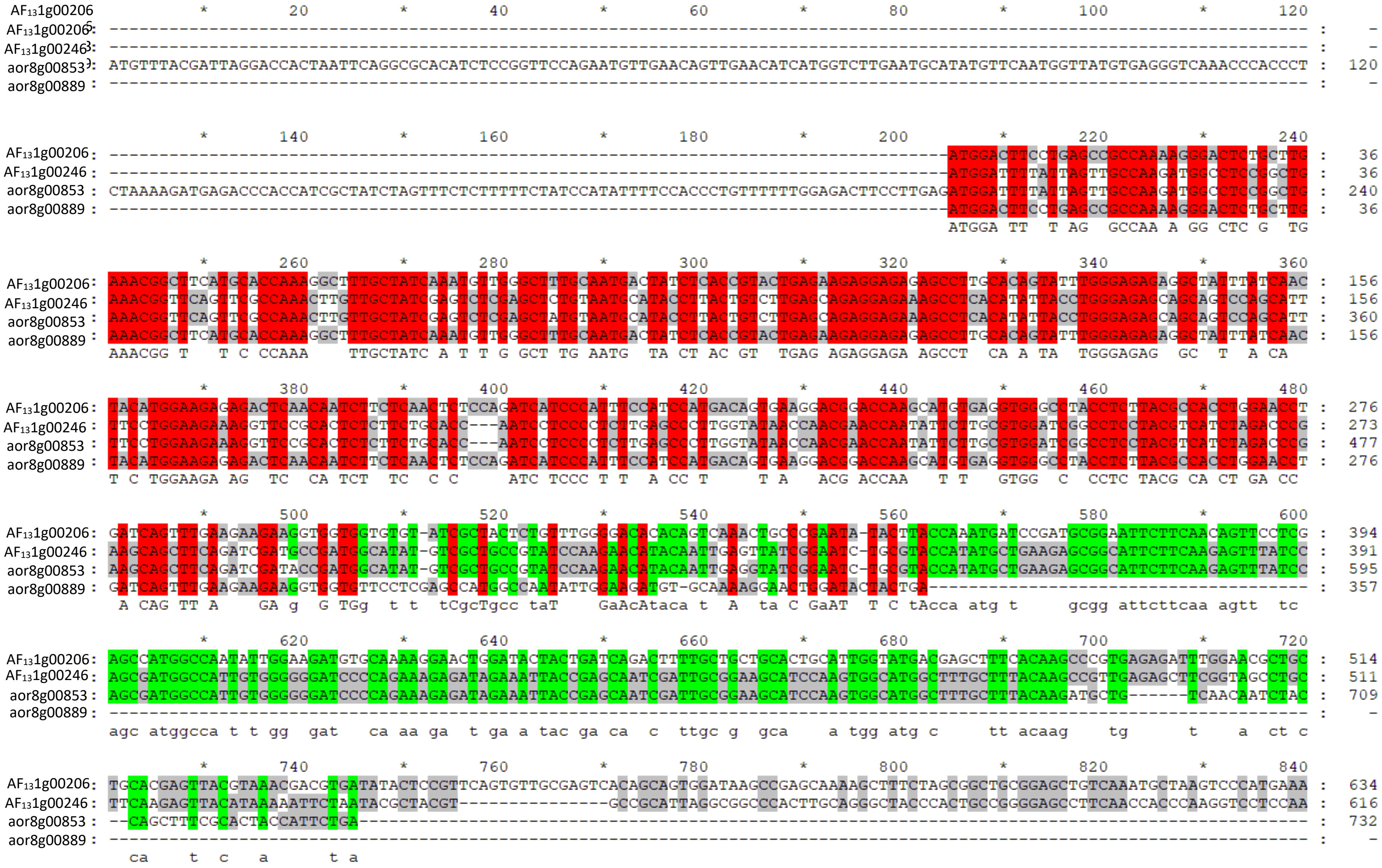

```
                         *        860         *        880         *        900         *        920         *        940         *        960
AF131g00206 : TGCAGCCAATACCAGACGACTCTGAAGGCAACAAACTTCTGGACACCAATGTTAATCGACGGGGTGAACGCCAAAATAGCCTTAGCTGCCAGTCGCAACTCCCGCAGGATTTGGCAGAAC :  754
AF131g00246 : TACAAGGGACTCAGGGCAGCTATGACGGCATGGTCATTAATGATGACATATTCCTACAAGGACGTCCAGCTACCCCACCACAGGTAAACCAGGGTTTGGCTGAAAGGTCCTGCTCCCAGA :  736
aor8g00853  : ------------------------------------------------------------------------------------------------------------------------ :    -
aor8g00889  : ------------------------------------------------------------------------------------------------------------------------ :    -

                         *        980         *       1000         *       1020         *       1040         *       1060         *       1080
AF131g00206 : GTATTTTGAATGAAAGCCCTCGGGCAAAGCAACTTTATTCATTGGAGCAACAGGGAACCGCGTCCAGTCATCAATTGGCTGAGGAGAATAACTTCTTTTCGAACCAGCTAAAAAGCCAGA :  874
AF131g00246 : TCCCCCAGACAGATCTGGATGTCGCGGAACAGCCCTGTGCTACCATTAGCTTTGGGGAGAATTCTCGCCACTGCTGTCTCAAAGAAGAGAAGGGCCTTTCGGACCAGCTAGAACATATAC :  856
aor8g00853  : ------------------------------------------------------------------------------------------------------------------------ :    -
aor8g00889  : ------------------------------------------------------------------------------------------------------------------------ :    -

                         *       1100         *       1120         *       1140         *       1160         *       1180         *       1200
AF131g00206 : AGGCAAATCCATCTAATGTTCACCGATGTTATATGCCAGGCTTCAAGTCGTATTCAGGGAAGATTCCGAGACAAAAGAGGAAGAATAAGGTTGTGGCCAACAGTGGCAGACATGGAATGG :  994
AF131g00246 : AGGTTGCTGATCCACAA---CCCAGAGAGCAGACCGGACACAAGAAAAGATGCAGAACAAGAGGCCGAACAGAACGGAGATTGACTGCTGCACGACGCGATAGCGCCAAGAGAAGGCAGA :  973
aor8g00853  : ------------------------------------------------------------------------------------------------------------------------ :    -
aor8g00889  : ------------------------------------------------------------------------------------------------------------------------ :    -

                         *
AF131g00206 : CGAGCATTAACTGGTAG : 1011
AF131g00246 : CAACTTTCGCC------ :  984
aor8g00853  : ----------------- :    -
aor8g00889  : ----------------- :    -
```

Supplemental Figure S2. The results of sequence alignment between the exons of strain AF13 and the introns of homologous genes in strain RIB40. This analysis aims to investigate the phenomenon of split genes with missing fragments in strain RIB40.

The gene correspondence for the four studied gene groups is specified as follows:A red background indicates that the four bases of two different species are identical. A green background indicates that three bases are the same. A gray background indicates that two bases are identical. a. The first group includes 2 genes from strain AF13 (AF131g00222, AF131g00232) and their 2 homologous counterpart genes in strain RIB40 (aor8g00868, aor8g00878); b. The second group consists of 2 genes from strain AF13 (AF131g00221, AF131g00233) and their 6 homologous counterpart genes in strain RIB40 (aor8g00865, aor8g00866, aor8g00867, aor8g00879, aor8g00880, aor8g00881); c. The third group comprises 2 genes from strain AF13 (AF131g00218, AF131g00240) and 3 homologous counterpart genes in strain RIB40 (aor8g00859, aor8g00860, aor8g00861); d. The fourth group contains 2 genes from strain AF13 (AF131g00206, AF131g00246) and their 2 homologous counterpart genes in strain RIB40 (aor8g00853, aor8g00889). The above alignment results clearly reveal the conservation differences and variation characteristics of intron sequences in RIB40, and further provide molecular-level evidence for clarifying the nature of missing fragments associated with split genes in strain RIB40.

a

Sequence	* 20 * 40 * 60 * 80 * 100 * 120	
AF$_{13}$1g00222 :	ATGACCGATAGCTCAAGAATCGATAGCTCAGGGACTCCTGCGAAGGCCCGGGGGAGCTGGCGGATAAACAAACGTAGACGACGCAGAGCCAGACATTCCCAACGAGTGACCGAGAGCAAT	: 120
AF$_{13}$1g00232 :	ATGACCGATAGCTCAAGAATCGATAGCTCAGGGACTCCTGCGAAGGCCCGGGGGAGCTGGCGGATAAACAAACGTAGACGACGCAGAGCCAGACATTCCCAACGAGTGACCGAGAGCAAT	: 120
aor8g00868(intron1):	--	: -
aor8g00878(intron1):	--	: -
aor8g00868(intron2):	--	: -
aor8g00878(intron2):	--	: -

Sequence	* 140 * 160 * 180 * 200 * 220 * 240	
AF$_{13}$1g00222 :	GGCCAAGAAGCTCAGGCCGATGACGCACATGGCGATGATACCCGTGCAGATGCCCTCGGCGAGCCTGATTCGGAGTCGGACAACGCTTTTGAGAAGATCATGCAGCGGAAGGAGATTCGT	: 240
AF$_{13}$1g00232 :	GGCCAAGAAGCTCAGGCCGATGACGCACATGGCGATGATACCCGTGCAGATGCCCTCGGCGAGCCTGATTCGGAGTCGGACAACGCTTTTGAGAAGATCATGCAGCGGAAGGAGATTCGT	: 240
aor8g00868(intron1):	--	: -
aor8g00878(intron1):	--	: -
aor8g00868(intron2):	--	: -
aor8g00878(intron2):	--	: -

Sequence	* 260 * 280 * 300 * 320 * 340 * 360	
AF$_{13}$1g00222 :	GAAGTCGCTACACAATTGGTTCACGGGAAGATGGAGGGGTACTGGACGGTCGTGGCCAACCTGTGCGTATTGCTCCAGATGGCCTGGAGGAAGGCTCCATTCCTTTTTCCACTGATGAAG	: 360
AF$_{13}$1g00232 :	GAAGTCGCTACACAATTGGTTCACGGGAAGATGGAGGGGTACTGGACGGTCGTGGCCAACCTGTGCGTATTGCTCCAGATGGCCTGGAGGAAGGCTCCATTCCTTTTTCCACTGATGAAG	: 360
aor8g00868(intron1):	--	: -
aor8g00878(intron1):	--	: -
aor8g00868(intron2):	--GTGCGTATTGCTCCAGATGGCCTGGAGGAAGGCTCCATTCCTTTTTCCACTGATGAAG	: 58
aor8g00878(intron2):	--GTGCGTATTGCTCCAGATGGCCTGGAGGAAGGCTCCATTCCTTTTTCCACTGATGAAG	: 58

Sequence	* 380 * 400 * 420 * 440 * 460 * 480	
AF$_{13}$1g00222 :	GAAGCGTTGGGACCCAATCGCGCACTGATTACCCTCATCCGGAAAGGACCCCATGCCGACATCATCCCGCAAAGCGTGCTTCCGTGGCCGTGCTGTGACGATGAACAACCTGGCTCGCCT	: 480
AF$_{13}$1g00232 :	GAAGCGTTGGGACCCAATCGCGCACTGATTACCCTCATCCGGAAAGGACCCCATGCCGACATCATCCCGCAAAGCGTGCTTCCGTGGCCGTGCTGTGACGATGAACAACCTGGCTCGCCT	: 480
aor8g00868(intron1):	--	: -
aor8g00878(intron1):	--	: -
aor8g00868(intron2):	--	: -
aor8g00878(intron2):	--	: -

Sequence	* 500 * 520 * 540 * 560 * 580 * 600	
AF$_{13}$1g00222 :	GCTGGTCCGGCGCGCCTCTCATCGCCGTCGCTCGCGGTTCCGAGCCCTGACGCGCCGATTGTTCAACCGCAGACGGCGGACGGTCCATCGCCGGCGACACCGGCGGCACCGTCGACGCTG	: 600
AF$_{13}$1g00232 :	GCTGGTCCGGCGCGCCTCTCATCGCCGTCGCTCGCGGTTCCGAGCCCTGACGCGCCGATTGCTCAACCGCAGACGGCGGACGGTCCATCGTCGGCGACACCGGCGGCACCGTCGACGCTG	: 600
aor8g00868(intron1):	--	: -
aor8g00878(intron1):	--	: -
aor8g00868(intron2):	--	: -
aor8g00878(intron2):	--	: -

```
                                    *      1220         *      1240         *      1260         *      1280         *      1300         *      1320
AF131g00222          : GGTCAGGTTGTACAAGCTGGCCATGCTTTTCAAACCGGTCAGGCTGGCCAGGCTCTTCAAACTGGCCATGCTTTTCAAACCGGTCAGGCTGGCCAGGCTCTTCAAACTGGCCAAACTAGT : 1320
AF131g00232          : GGTCAGGTTGTACAAGCGGGCCATGCTTTTCAAACCGGTCAGGCTGGTCAGGCTCTTCAAACTGGCCAAACTAGTCAAGTTGTCCAGACCGGTCAGGCTCTGCAAGCTGGTCAGGCTCTT : 1320
aor8g00868(intron1)  : ------------------------------------------------------------------------------------------------------------------------ :    -
aor8g00878(intron1)  : ------------------------------------------------------------------------------------------------------------------------ :    -
aor8g00868(intron2)  : ------------------------------------------------------------------------------------------------------------------------ :    -
aor8g00878(intron2)  : ------------------------------------------------------------------------------------------------------------------------ :    -

                                    *      1340         *      1360         *      1380         *      1400         *      1420         *      1440
AF131g00222          : CAAGTTGTCCAGACCGGTCAGGCTCTTCAAGCTGGTCAGGCCGTGCAAGCTGGTCAGACCGTTCAAACTGTTCAAGCTGGTCCGACTGCTCAAACTGCTCATACTGTTCAAGCTGTGCAA : 1440
AF131g00232          : CAAGCTG---------GTCAGGCTAGTCAAGCTGGTCAGGCTGTGCAAGCTGGTCAGACCGTTCAACCTGTTCAAGCTGGTCCGACTGCTCAAACTGCTCATACTGTTCAAGCTGTGCAA : 1431
aor8g00868(intron1)  : ------------------------------------------------------------------------------------------------------------------------ :    -
aor8g00878(intron1)  : ------------------------------------------------------------------------------------------------------------------------ :    -
aor8g00868(intron2)  : ------------------------------------------------------------------------------------------------------------------------ :    -
aor8g00878(intron2)  : ------------------------------------------------------------------------------------------------------------------------ :    -

                                    *      1460         *      1480         *      1500         *      1520         *      1540         *      1560
AF131g00222          : GCTGGTCTAGGGGCACCACCGCAACCTTCGTTTCCGATGGCCCACGGCCAACCTGCCGCTGCGGTCCCAAACGGCGGCGGCTTGCTGACCGCACCCCAGCCCGTTCAGCACTGGACGCCC : 1560
AF131g00232          : GCTGGTCTAGGGGCACCACCGCAACCTTCGTTTCCGATGGCCCACGGCCAACCTGCCGCTGCGGTCCCAAACGGCGGCGGCTTGCTGACCGCACCCCAGCCCGTTCAGCACTGGACGCCC : 1551
aor8g00868(intron1)  : ------------------------------------------------------------------------------------------------------------------------ :    -
aor8g00878(intron1)  : ------------------------------------------------------------------------------------------------------------------------ :    -
aor8g00868(intron2)  : ------------------------------------------------------------------------------------------------------------------------ :    -
aor8g00878(intron2)  : ------------------------------------------------------------------------------------------------------------------------ :    -

                                    *      1580         *      1600         *      1620         *      1640         *      1660         *      1680
AF131g00222          : GGCGTGGCGGGACTGCCGCAGGCAGCGACGCAAACCGC-CCCTCCAACGCAACCGGTAAACGCCGCGCTGTCCGCCCAACCAACAGACCCGTGTCAGGGAACGCTGGTGCATCCTGGGCA : 1679
AF131g00232          : GGCGTGGCGGGACTGCCGCAGGCAGCGACGCAAACCGC-CCCTCCAACGCAACCGGTAAACGCCGCGCTGTCCGCCCAACCAACAGACCCGTGTCAGGGAACGCTGGTGCATCCTGGGCA : 1670
aor8g00868(intron1)  : -GTATGTTCCGAATGCTGCGGGAATCGATGGCATCCTCACCCTCCATAG----------------------------------------------------------------------- :   48
aor8g00878(intron1)  : -GTATGTTCCGAATGCTGCGGGAATCGATGGCATCCTCACCCTCCATAG----------------------------------------------------------------------- :   48
aor8g00868(intron2)  : ------------------------------------------------------------------------------------------------------------------------ :    -
aor8g00878(intron2)  : ------------------------------------------------------------------------------------------------------------------------ :    -

                                    *      1700         *      1720         *      1740         *      1760         *      1780         *      1800
AF131g00222          : GCCGCAGGCATCAACCCAACCGTCCACCGGGCACCCTCCCGTCGAATCATCTCAGCAAACACCCTGGCCACCCCAGCAGCAGAATGACTCTGCGATGGACAGTGACCCGATGGCCAGCGA : 1799
AF131g00232          : GCCGCAGGCATCAACCCAACCGTCCACCGGGCACCCTCCCGTCGAACCATCTCAGCAAACACCCTGGCCACCCCAGCAGCAGAATGACTCTGCGATGGACAGTGACCCGATGGCCAGCGA : 1790
aor8g00868(intron1)  : ------------------------------------------------------------------------------------------------------------------------ :    -
aor8g00878(intron1)  : ------------------------------------------------------------------------------------------------------------------------ :    -
aor8g00868(intron2)  : ------------------------------------------------------------------------------------------------------------------------ :    -
aor8g00878(intron2)  : ------------------------------------------------------------------------------------------------------------------------ :    -
```

```
                              *        1820         *        1840         *        1860         *        1880         *        1900         *        1920
AF131g00222       : TGTCTCAGAGGATGCTCTGAACGCGGCCACGGAGGAAATCCGCCGACTGCAGGCGGACATGGCTCGCTATCGGGACGAGGTCGAGGAGCACATCCGATCCCTGAAACAGGCCCTGGACGA : 1919
AF131g00232       : TGTCTCAGAGGATGCTTTGAACGCGGCCACGGAGGAAATCCGCCGACTGCAAGCGGACATGGCTCGCTATCGGGATGAGGTCGAGGAGCACATCCGATCCCTGAAGCAGGCCCTGGACGA : 1910
aor8g00868(intron1): ------------------------------------------------------------------------------------------------------------------------ :    -
aor8g00878(intron1): ------------------------------------------------------------------------------------------------------------------------ :    -
aor8g00868(intron2): ------------------------------------------------------------------------------------------------------------------------ :    -
aor8g00878(intron2): ------------------------------------------------------------------------------------------------------------------------ :    -

                              *        1940         *        1960         *        1980         *        2000         *        2020         *        2040
AF131g00222       : GACCAATGAGCGGTACACAACCGAGATTCGGTACTATCCCCCGCCTCGGCCGAATGGTGCGGTCCGGGAGTCTCTGGGGCGACTATTGAATGCTGACCATGGTAACAGTGATATCCGCCA : 2039
AF131g00232       : GACCAATGAGCGGTACACAACCGAGATTCGGTACTATCCCCCGCCTCGGCCGAATGGTGCGGTCCGGGAGTCTCTGGGGCGACTATTGAATGCTGACCATGGTAACAGTGATATCCGCCA : 2030
aor8g00868(intron1): ------------------------------------------------------------------------------------------------------------------------ :    -
aor8g00878(intron1): ------------------------------------------------------------------------------------------------------------------------ :    -
aor8g00868(intron2): ------------------------------------------------------------------------------------------------------------------------ :    -
aor8g00878(intron2): ------------------------------------------------------------------------------------------------------------------------ :    -

                              *        2060         *        2080         *        2100         *        2120         *        2140
AF131g00222       : GTGTATGGCGAAGATGTGGGCGTATGAACAGGATATGAAAGCATGGACAACCAAGGCCGGAGAAACAATCAACAAGCTTTGCAAACGCCAGGGGAACTAA : 2139
AF131g00232       : GTGTATGGCGAAGATGTGGGCGTATGAACAGGATATGAAAGCATGGACAACCAAGGCCGGAGAAACAATCAACAAGCTTTGCAAACGCCAGGGGAACTGA : 2130
aor8g00868(intron1): ---------------------------------------------------------------------------------------------------- :    -
aor8g00878(intron1): ---------------------------------------------------------------------------------------------------- :    -
aor8g00868(intron2): ---------------------------------------------------------------------------------------------------- :    -
aor8g00878(intron2): ---------------------------------------------------------------------------------------------------- :    -
```

b

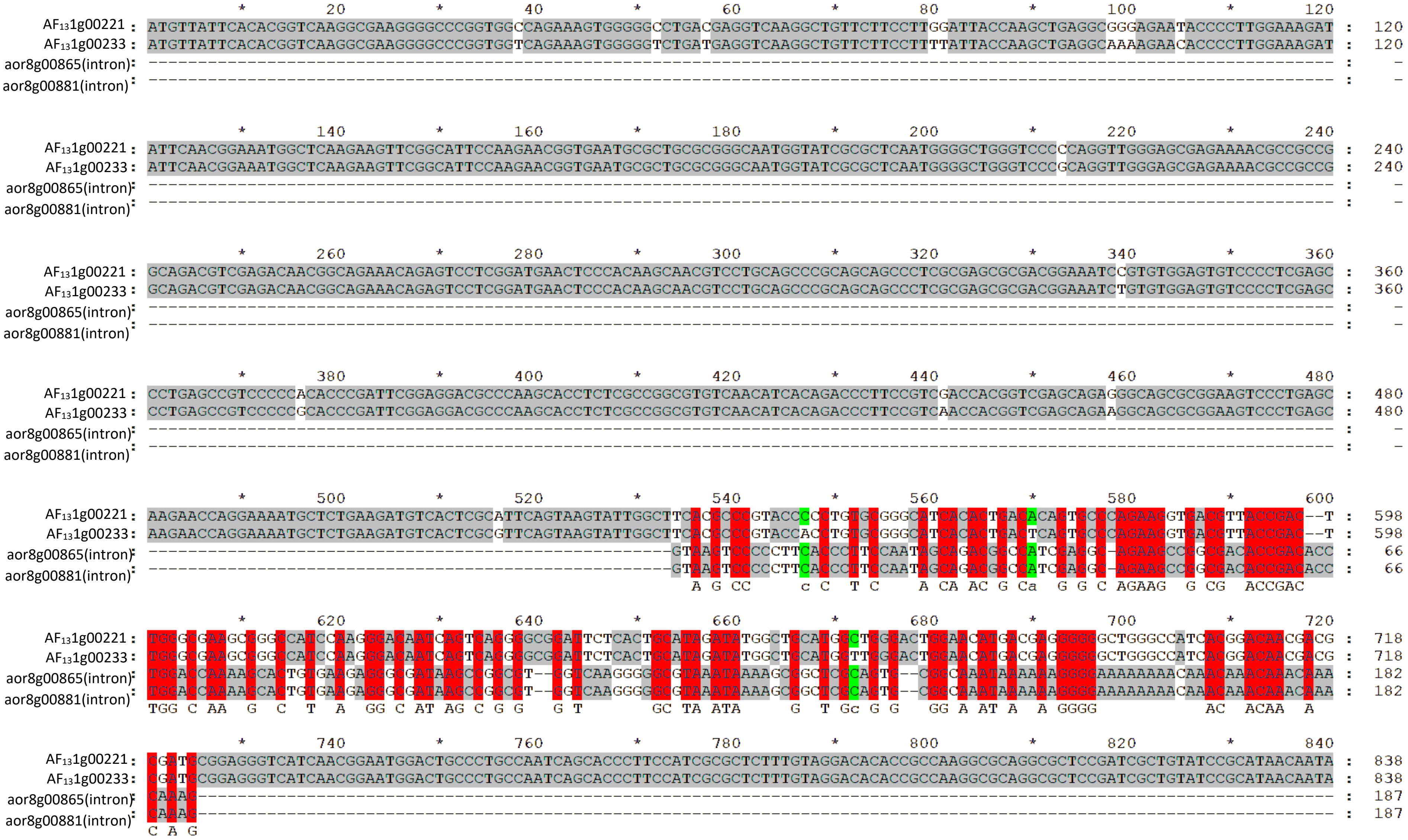
AF131g00221
AF131g00233
aor8g00865(intron)
aor8g00881(intron)

```
                              *         860         *         880         *         900         *         920         *         940         *         960
AF13 1g00221 :        CTGGCCGTCACCGGAAGCAGGGTTGGGCTCAACTGCACCTGTCCGGCGCGGCCACATCTCATCCGGTTATAGTCATCGAGAGCAGCCTCTCGCGCACGTCCCCGCCCGGGCCATCCCAGC : 958
AF13 1g00233 :        CTGGCCGTCACCGGAAGCAGGGTTGGGCTCAACTGCACCTGTCCGGCGCGGCCACATCTCATCCGGTTATAGTCATCGAGAGCAGCCTCTCGCGCACGTCCCCGCCCGGGCCATCCCAGC : 958
aor8g00865(intron) :  ------------------------------------------------------------------------------------------------------------------------ : -
aor8g00881(intron) :  ------------------------------------------------------------------------------------------------------------------------ : -

                              *         980         *        1000         *        1020         *        1040         *        1060         *        1080
AF13 1g00221 :        AGGAGCCACTCCGGCGATACCCCATCCCCCGCACTCGGGGACGCTCCTACGAGGATCTTCAGTCACTGCTGTACCGAGACGTCCTACTCCCGATCGTGGACACCGTGTGCGTCTTCGCAG : 1078
AF13 1g00233 :        AGGAGCCACTCCGGCGATACCCCATCCCCCGCACTCGGGGACGCTCCTACGAGGATCTTCAGTCACTGCTGTACCGAGACGTCCTACTCCCGATCGTGGACACCGTGTGCGTCTTCGCAG : 1078
aor8g00865(intron) :  ------------------------------------------------------------------------------------------------------------------------ : -
aor8g00881(intron) :  ------------------------------------------------------------------------------------------------------------------------ : -

                              *        1100         *        1120         *        1140         *        1160         *        1180         *        1200
AF13 1g00221 :        CCGACTGCGGCGGCATCCGTCACGTCCAACACTTGCTCGCATCGTGGGGCTCGCTACCGCCGACCGGTCTGGACGGCGCAGCGGCTTCCGTGCGTCCCCGTTGGGTGATTGTCCTGACAG : 1198
AF13 1g00233 :        CCGACTGCGGCGGCATCCGTCACGTCCAACACTTGCTCGCATCGTGGGGCTCGCTACCGCCGACCGGTCTGGACGGCGCAGCGGCTTCCGTGCGTCCCCGTTGGGTGATTGTCCTGACAG : 1198
aor8g00865(intron) :  ------------------------------------------------------------------------------------------------------------------------ : -
aor8g00881(intron) :  ------------------------------------------------------------------------------------------------------------------------ : -

                              *        1220         *        1240         *        1260         *        1280         *        1300         *        1320
AF13 1g00221 :        ACCCCGACGATGATGCAGTCCCGGCGGGAGGGATAGAAACCACGCTGCAGGCCACGGCTGTGCCCCATCTCGCGGGATCCGTCGCGGTTGTCGACCTGCGGCCCCGGCAGCCGTTGTCGC : 1318
AF13 1g00233 :        ACCCCGACGATGATGCAGTCCCGGCGGGAGGGATAGAAACCACGCTGCAGGCCACGGCTGTGCCCCATCTCGCGGGATCCGTCGCGGTTGTCGACCTGCGGCCCCGGCAGCCGTTGTCGC : 1318
aor8g00865(intron) :  ------------------------------------------------------------------------------------------------------------------------ : -
aor8g00881(intron) :  ------------------------------------------------------------------------------------------------------------------------ : -

                              *        1340         *        1360         *        1380         *        1400         *        1420         *        1440
AF13 1g00221 :        GGTTGAGCCGGTTCGAGCCTCTCCGGCGACGGCTGTCACTGGAGCTGGACGAGGTGCGCACGCTGCGTGCCAACGCACATCTCCTCTTCTCCGCCCTCCATCTGGAGTGGATCTTCCGGG : 1438
AF13 1g00233 :        GGTTGAGCCGGTTCGAGCCTCTCCGGCGACGGCTGTCACTGGAGCTGGACGAGGTGCGCACGCTGCGTGCCAACGCACATCTCCTCTTCTCCGCCCTCCATCTGGAGTGGATCTTCCGGG : 1438
aor8g00865(intron) :  ------------------------------------------------------------------------------------------------------------------------ : -
aor8g00881(intron) :  ------------------------------------------------------------------------------------------------------------------------ : -

                              *        1460         *        1480         *        1500         *        1520         *        1540         *        1560
AF13 1g00221 :        GACTGCTGCGGCACGTCGCGCAAGGCCCTGCGTCGCCATTCAATTGCATCCAAGCGTGCCGTCCCCAGCACTTGGACGCTGATGAGGTCGCTCAATACCTCGGAATCTTCCTGCAGCTCG : 1558
AF13 1g00233 :        GACTGCTGCGGCACGTCGCGCAAGGCCCTGCGTCGCCATTCAATTGCATCCAAGCGTGCCGTCCCCAGCACTTGGACGCTGATGAGGTCGCTCAATACCTCGGAATCTTCCTGCAGCTCG : 1558
aor8g00865(intron) :  ------------------------------------------------------------------------------------------------------------------------ : -
aor8g00881(intron) :  ------------------------------------------------------------------------------------------------------------------------ : -

                              *        1580         *        1600         *        1620         *        1640         *        1660         *        1680
AF13 1g00221 :        CCGAGAAGACCCAGATAGCGGACCCATTCATGGCAACCTTCATTGCGTCGGCGTTCCTTATGGATGCATATCCGCCCGGAATGCACAGATTCGATCCCGTCGTGACGTTCCGCACCTTCT : 1678
AF13 1g00233 :        CCGAGAAGACCCAGATAGCGGACCCATTCATGGCAACCTTCATTGCGTCGGCGTTCCTTATGGATGCATATCCGCCCGGAATGCACAGATTCGATCCCGTCGTGACGTTCCGCACCTTCT : 1678
aor8g00865(intron) :  ------------------------------------------------------------------------------------------------------------------------ : -
aor8g00881(intron) :  ------------------------------------------------------------------------------------------------------------------------ : -
```

```
                             *       1700         *       1720         *       1740         *       1760         *       1780         *       1800
AF131g00221        : ACGCCGGGCCCTGTAAAGCCGCATGCGCCGCATCTCCCTCCGCCAGCGTTCGCCGTCTCCACCGTGCCGTCGAACGCGAGTTCGTGTCGCTCTTTTCCACCCTCGCCGTGGCCGGCCGGT : 1798
AF131g00233        : ACGCCGGGCCCTGTAAAGCCGCATGCGCCGCATCTCCCTCCGCCAGCGTTCGCCGTCTCCACCGTGCCGTCGAACGCGAGTTCGTGTCGCTCTTTTCCACCCTCGCCGTGGCCGGCCGGT : 1798
aor8g00865(intron) : ------------------------------------------------------------------------------------------------------------------------ :    -
aor8g00881(intron) : ------------------------------------------------------------------------------------------------------------------------ :    -

                             *       1820         *       1840         *       1860         *       1880         *       1900         *       1920
AF131g00221        : CCGCCCAGATCCGACGGGAGGTGTTCCGCAACGGCCAGTCGACCTGGTCGACGCTCCATTCGAATCGGGTGTGCCTGTTCTGTCTGCAGCGGCCCCCCGAGCATGTGCTGCCCTGCAGGC : 1918
AF131g00233        : CCGCCCAGATCCGACGGGAGGTGTTCCGCAACGGCCAGTCGACCTGGTCGACGCTCCATTCGAATCGGGTGTGCCTGTTCTGTCTGCAGCGGCCCCCCGAGCATGTGCTGCCCTGCAGGC : 1918
aor8g00865(intron) : ------------------------------------------------------------------------------------------------------------------------ :    -
aor8g00881(intron) : ------------------------------------------------------------------------------------------------------------------------ :    -

                             *       1940         *       1960         *       1980         *       2000         *       2020         *       2040
AF131g00221        : ATGCCCTCTGCGACGTCTGCGCGTGCATTTTGGGCCAACGTGCCGCTGGCGCGGAATATCACGTCGAGCTAACCGAGTGTCCCGCGTGTCAGGCGTCGTTCTCCCTCACCGTCCGTTTGC : 2038
AF131g00233        : ATGCCCTCTGCGACGTCTGCGCGTGCATTTTGGGCCAACGTGCCGCTGGCGCGGAATATCACGTCGAGCTAACCGAGTGTCCCGCGTGTCAGGCGTCGTTCTCCCTCACCGTCCGTTTGC : 2038
aor8g00865(intron) : ------------------------------------------------------------------------------------------------------------------------ :    -
aor8g00881(intron) : ------------------------------------------------------------------------------------------------------------------------ :    -

                             *       2060         *       2080         *       2100         *       2120         *       2140         *       2160
AF131g00221        : TCCCTCCAACCAAACGGCCTACCATCCTGGTCCTGGACGGAGGCGGCACCCGCGGCGTCGTCACGCTGGGGTTCTTGAAGGCCCTCGAGGATCAGATCGGCCGCACGCGAGGACTGCGTG : 2158
AF131g00233        : TCCCTCCAACCAAACGGCCTACCATCCTGGTCCTGGACGGAGGCGGCACCCGCGGCGTCGTCACGCTGGGGTTCTTGAAGGCCCTCGAGGATCAGATCGGCCGCACGCGAGGACTGCGTG : 2158
aor8g00865(intron) : ------------------------------------------------------------------------------------------------------------------------ :    -
aor8g00881(intron) : ------------------------------------------------------------------------------------------------------------------------ :    -

                             *       2180         *       2200         *       2220         *       2240         *       2260         *       2280
AF131g00221        : AGGCATTCGACCTCACGCTGGGAACCAGTGTTGGCGCCATCATCGCATCCGACGTAATGGTCTGCGGTGCCAACGTGGCCGACACCCGTCCGAAGTTCGATACCCTGGCACGTCAGATCT : 2278
AF131g00233        : AGGCATTCGACCTCACGCTGGGAACCAGTGTTGGCGCCATCATCGCATCCGACGTAATGGTCTGCGGTGCCAACGTGGCCGACACCCGTCCGAAGTTCGATACCCTGGCACGTCAGATCT : 2278
aor8g00865(intron) : ------------------------------------------------------------------------------------------------------------------------ :    -
aor8g00881(intron) : ------------------------------------------------------------------------------------------------------------------------ :    -

                             *       2300         *       2320         *       2340         *       2360         *       2380         *       2400
AF131g00221        : TTTCCCGACGGCCCCTCTGGCAGACGATCTTGGGACAATCGTGGGGCTGGGTGACGGCCTGGATGGCCGACAGCCGGTACGACTCGGCCGTGTTGGACCGAACTGTGCAGGATGGCTTTG : 2398
AF131g00233        : TTTCCCGACGGCCCCTCTGGCAGACGATCTTGGGACAATCGTGGGGCTGGGTGACGGCCTGGATGGCCGACAGCCGGTACGACTCGGCCGTGTTGGACCGAACTGTGCAGGATGGCTTTG : 2398
aor8g00865(intron) : ------------------------------------------------------------------------------------------------------------------------ :    -
aor8g00881(intron) : ------------------------------------------------------------------------------------------------------------------------ :    -

                             *       2420         *       2440         *       2460         *       2480         *       2500         *       2520
AF131g00221        : GCCGCGACCGCCGACTGTTCGATACCACGAAACCCTTGGTATCCGGCATCCGAGTGGCTCTAACAGCCAGCCAGGTCGAAGACGGGTCGCTCTGTCTGTTCTCCAACTATCGAGCGGCGG : 2518
AF131g00233        : GCCGCGACCGCCGACTGTTCGATACCACGAAACCCTTGGTATCCGGCATCCGAGTGGCTCTAACAGCCAGCCAGGTCGAAGACGGGTCGCTCTGTCTGTTCTCCAACTATCGAGCGGCGG : 2518
aor8g00865(intron) : ------------------------------------------------------------------------------------------------------------------------ :    -
aor8g00881(intron) : ------------------------------------------------------------------------------------------------------------------------ :    -
```

```
                               *        2540         *        2560         *        2580         *        2600         *        2620         *        2640
AF131g00221       : GCCGTCCTCGGATGTCGTCCGCCTATAGAGCACTAGTGCCAGAGCAGGAGCCATTTTTGTGGGAAATTGCCCGGTGCTGCGTGGCTGCCTTAGGGTACGTATCTCGGGACATGCTGGAAG : 2638
AF131g00233       : GCCGTCCTCGGATGTCGTCCGCCTATAGAGCACTAGTGCCAGAGCAGGAGCCATTTTTGTGGGAAATTGCCCGGTGCTGCGTGGCTGCCTTAGGGTACGTATCTCGGGACATGCTGGAAG : 2638
aor8g00865(intron): ------------------------------------------------------------------------------------------------------------------------ :    -
aor8g00881(intron): ------------------------------------------------------------------------------------------------------------------------ :    -

                               *        2660         *        2680         *        2700         *        2720         *        2740         *        2760
AF131g00221       : TGAGTCGGGACAACGACCGAGCCACCGCGTTAACCTACCCCAACAGGTATTTCACCCCTAAACGACTCCTGGGCCTTGGGACCTTCCAAGACGGTGGAGTTCGGGCGAATTGTCCGCTTC : 2758
AF131g00233       : TGAGTCGGGACAACGACCGAGCCACCGCGTTAACCTACCCCAACAGGTATTTCACCCCTAAACGACTCCTGGGCCTTGGGACCTTCCAAGACGGTGGAGTTCGGGCGAATTGTCCGCTTC : 2758
aor8g00865(intron): ------------------------------------------------------------------------------------------------------------------------ :    -
aor8g00881(intron): ------------------------------------------------------------------------------------------------------------------------ :    -

                               *        2780         *        2800         *        2820         *        2840         *        2860         *        2880
AF131g00221       : GAACCGCTCTCCGCGAGAGTGAGATCATCTGGCCGACAGCCAAACGACCCGACCTCGTGGTCAGCATCGGCACCGGCTATACGTCGGCCGACGACGGACCTGTCCACCTGCGGCGAGAAA : 2878
AF131g00233       : GAACCGCTCTCCGCGAGAGTGAGATCATCTGGCCGACAGCCAAACGACCCGACCTCGTGGTCAGCATCGGCACCGGCTATACGTCGGCCGACGACGGACCTGTCCACCTGCGGCGAGAAA : 2878
aor8g00865(intron): ------------------------------------------------------------------------------------------------------------------------ :    -
aor8g00881(intron): ------------------------------------------------------------------------------------------------------------------------ :    -

                               *        2900         *        2920         *        2940         *        2960         *        2980         *        3000
AF131g00221       : GTTTCGTCGAACGCGGCCTGCGGGCCTTTCTGTCCTCGCCGGCGGTCGACGGGCGACGCGGCTGGCAGGACGCGCACGACAGCATCCCCGAGAGTGTCAAGCAGGAGACCTTCCGCCTGG : 2998
AF131g00233       : GTTTCGTCGAACGCGGCCTGCGGGCCTTTCTGTCCTCGCCGGCGGTCGACGGGCGACGCGGCTGGCAGGACGCGCACGACAGCATCCCCGAGAGTGTCAAGCAGGAGACCTTCCGCCTGG : 2998
aor8g00865(intron): ------------------------------------------------------------------------------------------------------------------------ :    -
aor8g00881(intron): ------------------------------------------------------------------------------------------------------------------------ :    -

                               *        3020         *        3040         *        3060         *        3080         *        3100         *        3120
AF131g00221       : ACCGGGTTGTCTCCGGCGCGCTACCTGAACTGGACGACGTGCGCGCCCTTCACGAACTAGGTGAGTATGAATACCACATCCCCGACGAGCTGACCCAGGCTTGGCTGGCCAAGTCGTTCT : 3118
AF131g00233       : ACCGGGTTGTCTCCGGCGCGCTACCTGAACTGGACGACGTGCGCGCCCTTCACGAACTAGGTGAGTATGAATACCACATCCCCGACGAGCTGACCCAGGCTTGGCTGGCCAAGTCGTTCT : 3118
aor8g00865(intron): ------------------------------------------------------------------------------------------------------------------------ :    -
aor8g00881(intron): ------------------------------------------------------------------------------------------------------------------------ :    -

                               *        3140         *        3160         *        3180         *        3200         *        3220         *        3240
AF131g00221       : TCTTCGAGCTGGACGAGGAGCCACGGTTCACCCGCGGGCACTACGAGTGCCGCGGGTCGATCCTCTGTTGTAAGTATGGCGCCGCCGGTCTTGTTGAGCAGATCCTGTCCCACTTCCCCG : 3238
AF131g00233       : TCTTCGAGCTGGACGAGGAGCCACGGTTCACCCGCGGGCACTACGAGTGCCGCGGGTCGATCCTCTGTTGTAAGTATGGCGCCGCCGGTCTTGTTGAGCAGATCCTGTCCCACTTCCCCG : 3238
aor8g00865(intron): ------------------------------------------------------------------------------------------------------------------------ :    -
aor8g00881(intron): ------------------------------------------------------------------------------------------------------------------------ :    -

                               *        3260         *        3280         *        3300         *        3320         *        3340         *        3360
AF131g00221       : GAGCGCGCTTCGCGCTCGACGACGGCAGTGACCTGGGTGCCGTTGATCACGACCAGGGCTGTCTCACCTGTGGCTATTACCGCAAGCGAGTCACCTTCCGCGTGTCGAGCATCCACCAGA : 3358
AF131g00233       : GAGCGCGCTTCGCGCTCGACGACGGCAGTGACCTGGGTGCCGTTGATCACGACCAGGGCTGTCTCACCTGTGGCTATTACCGCAAGCGAGTCACCTTCCGCGTGTCGAGCATCCACCAGA : 3358
aor8g00865(intron): ------------------------------------------------------------------------------------------------------------------------ :    -
aor8g00881(intron): ------------------------------------------------------------------------------------------------------------------------ :    -
```

```
                             *        3380         *        3400         *        3420         *        3440         *        3460         *        3480
AF13 1g00221 :         CCCTGCAGCTAGGCATTCGCGGGGTCGCCAGGCACAGGGCCATCGGTGGCTTCCCCACTTCCATCCACGATCTCCTCCATGAACAGCAAGCCGACTGCCCGTTTGGGCGAGCTGACCATC : 3478
AF13 1g00233 :         CCCTGCAGCTAGGCATTCGCGGGGTCGCCAGGCACAGGGCCATCGGTGGCTTCCCCACTTCCATCCACGATCTCCTCCATGAACAGCAAGCCGACTGCCCGTTTGGGCGAGCTGACCATC : 3478
aor8g00865(intron) :   ------------------------------------------------------------------------------------------------------------------------ :    -
aor8g00881(intron) :   ------------------------------------------------------------------------------------------------------------------------ :    -
```

```
                             *        3500         *        3520         *        3540         *        3560         *        3580
AF13 1g00221 :         GCACTAATTTTTGGCCGCCGGTGCGCCGGTGCTACTGTCCCGCGCGGAAGCGATCTCACACATCGATGCAGACTGAGCGCGCGACCAAGAGGCGACGGCTCTAG : 3582
AF13 1g00233 :         GCACTAATTTTTGGCCGCCGGTGCGCCGGTGCTACTGTCCCGCGCGGAAGCGATCTCACACATCGATGCAGACTGAGCGCGCGACCAAGAGGCGACGGCTCTAG : 3582
aor8g00865(intron) :   -------------------------------------------------------------------------------------------------------- :    -
aor8g00881(intron) :   -------------------------------------------------------------------------------------------------------- :    -
```

(Sequence names: $AF_{13}1g00221$, $AF_{13}1g00233$)

C

```
                                 *        20         *        40         *        60         *        80         *       100         *       120
AF$_{13}$1g00218 :     ATGGAATACGACACGCTGGACTCCCCAGAGCCGCGAGCAACCCCCAATCAGCCCATCCACGCTGGCGTGGAGGGACTGACCGCTCCGGAGTTGACGGCGCCCCGCAGCATTGCCCTGGGC : 120
AF$_{13}$1g00240 :     ATGGAACACGATACGCAGGACTCCCCAGAGCCGCGAGTAACCCCCAATCAGCCCATCCACGCTGGCGTGGAGGGACCGACCGCTCCGGAGTTGACGGCGCCCCGCAGCATTGCCCTGGGC : 120
aor8g00861(intron) : ------------------------------------------------------------------------------------------------------------------------ : -
                     atggaa acga acgc ggactccccagagccgcgag aacccccaatcagcccatccacgctggcgtggagggac gaccgctccggagttgacggcgccccgcagcattgccctgggc

                                 *       140         *       160         *       180         *       200         *       220         *       240
AF$_{13}$1g00218 :     TTGACCCGTGACTATGCGGCCAACTGGACGGTTCCCGATGCCCTGCGGGAGCTCTATCAAAACTGGAAGGACGCCATCCTCCAGACCCACTCGATCAGCCTGCTCGAGTTTGCCCCCCGG : 240
AF$_{13}$1g00240 :     TTGACCCGTGACTATGCGGCCAACTGGACGGTTCCCGATGCCCTGCGGGAGCTCTATCAAAACTGGAAGGACGCTATCCTCCAGACCCACTCGATCAGTCTGCTCGAGTTTGCACCCCGG : 240
aor8g00861(intron) : ------------------------------------------------------------------------------------------------------------------------ : -
                     ttgacccgtgactatgcggccaactggacggttcccgatgccctgcgggagctctatcaaaactggaaggacgc atcctccagacccactcgatcag ctgctcgagtttgc ccccgg

                                 *       260         *       280         *       300         *       320         *       340         *       360
AF$_{13}$1g00218 :     GTGACGGCAACGTCGGACGCCATCACGATTGTCGTGGAGGGGTTGCAGCCACCCTTCGGCGGGGGCGGCGGAGGGCCCCTGGTCCGGAGGATCTTGGGATATATTCGCTTCAACAAGCCT : 360
AF$_{13}$1g00240 :     GTGTCGGCAACGTCGGACGCCATCACGATTGTCGTGGAGGGGTTGCAACCATCCTTCGGCGGGGGCGGCGGAGGGCCCCTGGTCCGAAGGATCTTGGGATATATTCGCTTCAACAAGCCC : 360
aor8g00861(intron) : ------------------------------------------------------------------------------------------------------------------------ : -
                     gtg cggcaacgtcggacgccatcacgattgtcgtggaggggttgca cca ccttcggcgggggcggcggagggcccctggtccg aggatcttgggatatattcgcttcaacaagcc

                                 *       380         *       400         *       420         *       440         *       460         *       480
AF$_{13}$1g00218 :     CAAGGCAGTGCGGAGTTCACCAATTTTAGATCCTCTCTGGACCCGCAATGCTTGGCGATCGGTCACAGCAGCAAGAAGCAAGACGAGCGCCTGGCCGGTGGTCACGGAGAGGGGCTGAAG : 480
AF$_{13}$1g00240 :     CAAGGCAGTGCGGAGTTCACCAATTTTAGATCCTCTCTGGACCCGCAATGCTTGGCGATCGGTCACAGCAGCAAGAAGCAAGACGAGCGCCTGGCCGGTGGTCACGGAGAGGGGCTGAAG : 480
aor8g00861(intron) : ------------------------------------------------------------------------------------------------------------------------ : -
                     caaggcagtgcggagttcaccaattttagatcctctctggacccgcaatgcttggcgatcggtcacagcagcaagaagcaagacgagcgcctggccggtggtcacggagaggggctgaag

                                 *       500         *       520         *       540         *       560         *       580         *       600
AF$_{13}$1g00218 :     ATCGCCGCGTTGGTCCTCTCGCGGGCGGAGCATCACGTCAAGATCTCCGCGAGCGGGACCTACTGGAACTTTGGCTTCAACGGCCACTCGAAATCGAACTTTTACTGCCGGCTGTCACCG : 600
AF$_{13}$1g00240 :     ATCGCCGCGTTGGTCCTCTCGCGGGCGGAGCATCACGTCAAGATCTCCGCGAGCGGGACCTACTGGAACTTTGGCTTCAACGGCCACTCGAAATCGAACTTTTACTGCCGGCTTTCACCG : 600
aor8g00861(intron) : ------------------------------------------------------------------------------------------------------------------------ : -
                     atcgccgcgttggtcctctcgcgggcggagcatcacgtcaagatctccgcgagcgggacctactggaactttggcttcaacggccactcgaaatcgaacttttactgccggct tcaccg

                                 *       620         *       640         *       660         *       680         *       700         *       720
AF$_{13}$1g00218 :     GCCAAGGCGAAGCGGGATCCCGGGCTCGCCAGCGACGGCATTCTGCTCCCCCGGCTGACCGCCGAGGTCGGACGGGACGTCTCGGTGCTGGTCGAGAAAGGCCAGAAGGGCCAGCGGTTG : 720
AF$_{13}$1g00240 :     GCCAAGGCGAAGCGGGATCCCGGGCCCGCCAGCGACGGCATTCTGCTCCCCCGGCTGACCGCCGAGGTCGGACGGGACGTCTCGGTGCTGGTCGAGAAAGGCCAGAAGGGCCAGCGGTTG : 720
aor8g00861(intron) : ------------------------------------------------------------------------------------------------------------------------ : -
                     gccaaggcgaagcgggatcccgggc cgccagcgacggcattctgctcccccggctgaccgccgaggtcggacgggacgtctcggtgctggtcgagaaaggccagaagggccagcggttg

                                 *       740         *       760         *       780         *       800         *       820         *       840
AF$_{13}$1g00218 :     TCGCTGGAAGATTTCAAGGCCTGGATGTGGGACACGGTCGATCTCCACGCGCCGTCGAGCTCGGTCCGGACGCCCGTTGGCGACCTGCTGCTGGGGCCGGCACACCGGGGCCGTCTGTAT : 840
AF$_{13}$1g00240 :     TCGCTGGAAGATTTCAAGGCCTGGATGTGGGACACGGTCGATCTCCACGCGCCGTCGAGCTCGGTTCGGACGCCCGTTGGCGACCTGCTGCTGGGGCCGGCACACCGGGGCCGTCTGTAT : 840
aor8g00861(intron) : ------------------------------------------------------------------------------------------------------------------------ : -
                     tcgctggaagatttcaaggcctggatgtgggacacggtcgatctccacgcgccgtcgagctcggt cggacgcccgttggcgacctgctgctggggccggcacaccggggccgtctgtat
```

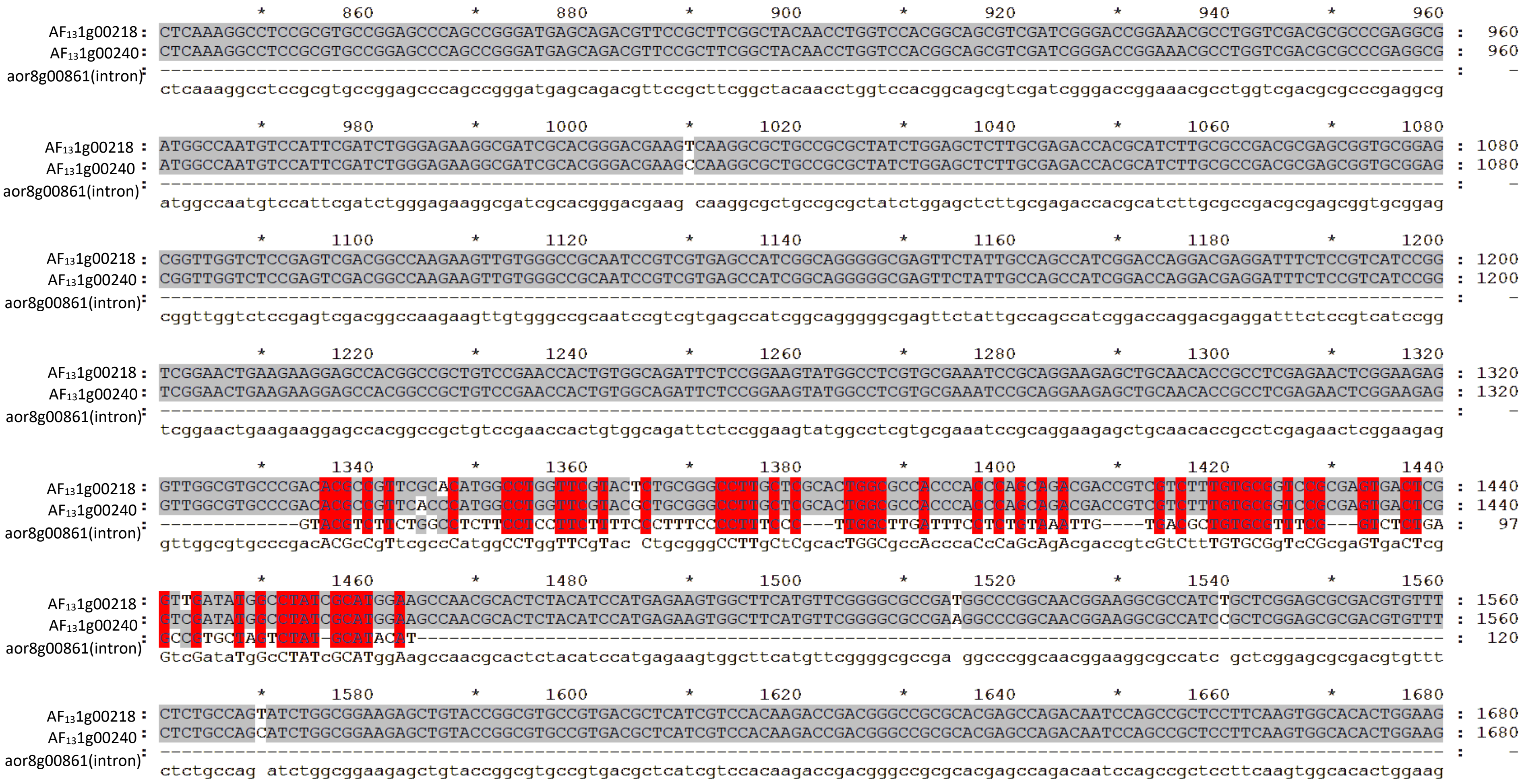
AF131g00218
AF131g00240
aor8g00861(intron)

* 1700 * 1720 * 1740 * 1760 * 1780 * 1800

AF$_{13}$1g00218 : TTGCACGAGATGCCACGCCGGATTGAGGTGTACGCCGCCGCCGCTGACGACGGACCGAGGGCGACACAGGTCTCGTTCTACACGGGCCACAGCCTCCTGTTCACTAAGCTATGCGGGACG : 1800
AF$_{13}$1g00240 : TTGCACGAGATGCCACGCCAGATTGAGGTGTACGCCGCCGCCGCTGACGACGGCCCGAGGACGACACAGGTCTCGTTCTACACGGGCCACAGCCTCCTGTTCACTAAGCTGTGCGGGACG : 1800
aor8g00861(intron) : -- : -
ttgcacgagatgccacgcc gattgaggtgtacgccgccgccgctgacgacgg ccgagg cgacacaggtctcgttctacacgggccacagcctcctgttcactaagct tgcgggacg

* 1820 * 1840 * 1860 * 1880 * 1900 * 1920

AF$_{13}$1g00218 : CAAGTGTCGTATCTGGTCGTGCTACATGGGCCCAACTGCGTCGCGCAGTCGGTGGATCACCTGCTGTACGACCCCAGCCGGGACTGCTGTCTGTGCCCGCGGCAGGTGGTGCCCCTGAGC : 1920
AF$_{13}$1g00240 : CAAGTGTCGTATCTGGTCGTGCTACATGGGCCCAACTGCGTCGCGCAGTCGGTGGATCACCTGCTGTACGACCCCAGCCGGGACTGCTGTCTGTGCCCGCGGCAGGTGGTGCCCCTGAGC : 1920
aor8g00861(intron) : -- : -
caagtgtcgtatctggtcgtgctacatgggcccaactgcgtcgcgcagtcggtggatcacctgctgtacgaccccagccgggactgctgtctgtgcccgcggcaggtggtgcccctgagc

* 1940 * 1960 * 1980 * 2000 * 2020 * 2040

AF$_{13}$1g00218 : ATGCGACGAGCTGTGTTCGAGGATCCTGGCCACGGGCCCTGGGTTCCCATGGTGGTGAAGATGTCGGTTCGTGACGGCCTTGTCGGCGCCATGTCCTCCCCAGGGGGCCATCATTGGCCG : 2040
AF$_{13}$1g00240 : ATGCGACGAGCTGTGTTCGAGGATCCTGGCCACGGGCCCTGGGTTCCCATGGTGGTGAAAATGTCGGTTCGTGACGGCCTTGTCGGCGCCATGTCCTCCCCAGGGGGCCATCATTGGCCG : 2040
aor8g00861(intron) : -- : -
atgcgacgagctgtgttcgaggatcctggccacgggccctgggttcccatggtggtgaa atgtcggttcgtgacggccttgtcggcgccatgtcctccccagggggccatcattggccg

* 2060 * 2080 * 2100 * 2120 * 2140 * 2160

AF$_{13}$1g00218 : CTTCTTCCGAAAGCGCAGGACGGAGCGTTGGTGGCGATTGCGCCTCCTTCCCACTCTTCGTCATCGGAGACTGCAGCTCCCCGCGGGACCGTCCCGGTGGATCGTCCGCCCACGGTCTGT : 2160
AF$_{13}$1g00240 : CTTCTTCCGAAAGCGCAGGACCGAGCGTTGGTGGCGATTGCGCCTCCTTCCCACTCTCCGTCATCGGAGACTGCAGCTCCCCGCGGGACCGTCCCGGTGGATCGTCCGCCCACGGTCAGT : 2160
aor8g00861(intron) : -- : -
cttcttccgaaagcgcaggac gagcgttggtggcgattgcgcctccttcccactct cgtcatcggagactgcagctccccgcgggaccgtcccggtggatcgtccgcccacggtc gt

* 2180 * 2200 * 2220 * 2240 * 2260 * 2280

AF$_{13}$1g00218 : GCTGCCTTGGATCCTGTGCTCACCACCCCGACGGCTGCCAGCCAGGAGAACCAAGAAACGACAGCCGCGTCGCAGCTGGCCGCTAGGCCACTTGAACTGGAAACGGGCGCGCAGACGACG : 2280
AF$_{13}$1g00240 : GCTGCCTTGGATCCTGTGCTCACCACTCCGACGGCTGCCAGCCAGGAGAACCAAGAAACGACAGCCGCGTCGCAGCTGGCCGCTAGGCCGCTTGAACTGGAAACGGGCGCGCAGACGACG : 2280
aor8g00861(intron) : -- : -
gctgccttggatcctgtgctcaccac ccgacggctgccagccaggagaaccaagaaacgacagccgcgtcgcagctggccgctaggcc cttgaactggaaacgggcgcgcagacgacg

* 2300 * 2320 * 2340 * 2360 * 2380 * 2400

AF$_{13}$1g00218 : GCATCGGACACCGCATCAGACACCGCATCAGACACCGCATCAGACACCACAGCGCCGGTCCCTCTCGTATCAGTGGTGGAATGCCTCGC------CCCGGATGACCATCCTGAGTCCGCG : 2394
AF$_{13}$1g00240 : GCATCGGACACCGCATCAGACACCGCATCAGACACCGCA------------GCACCGGTCCCTCTCGTATCAGTGGTGGAATGCCTCGCCCCCCACCCGGATGACCATCCCGAGTCCGCG : 2388
aor8g00861(intron) : -- : -
gcatcggacaccgcatcagacaccgcatcagacaccgca   gc ccggtccctctcgtatcagtggtggaatgcctcgc   cccggatgaccatcc gagtccgcg

* 2420 * 2440 * 2460 * 2480 * 2500 * 2520

AF$_{13}$1g00218 : TCAAATGCGCTGCCCTCGACCATCTCCGGGAATATGACCTCTGCGGATGGTGCGTGGTGGCAGACCTGGCCAGAACCACCCCGGGATGTCCAAACGTCACCGAGCGCTGCGGATCAGAAC : 2514
AF$_{13}$1g00240 : TCTGGTGCGCTGCCCTCGACCATCTCCCGGAATATGACCTCTGCGGATGGTGCGTGGTGGCAGACATGGCCAGAACCGCCCCGGGATGTCCAAACGTCACCGAGCGTTGCGGATCACAAC : 2508
aor8g00861(intron) : -- : -
tc   tgcgctgccctcgaccatctcc ggaatatgacctctgcggatggtgcgtggtggcagac tggccagaacc ccccgggatgtccaaacgtcaccgagcg tgcggatca aac

```
                              *      2540         *      2560         *      2580         *      2600         *      2620         *      2640
AF131g00218        : ATTGGACGCTTTCATTCTAGCTGGGTCTCCTCCCTCTCGCCGGATCTGCTCCGCCGTTTCCCTACAAACCCCTTGCTCTCCCGTTGCACGAGGGGACGCCGTGGTTTAACAATCCTGCAG : 2634
AF131g00240        : ATTGGACGCTTTCA---------------------------------------------------------------------------------------------------------G : 2523
aor8g00861(intron) : ------------------------------------------------------------------------------------------------------------------------ :    -
                     attggacgctttca                                                                                                         g

                              *      2660         *      2680         *      2700         *      2720         *      2740         *      2760
AF131g00218        : GTCCAGGAATCGCATCGGTCATGCGAGCCACCCCAGGGCCTCTCGAAAGGGCAATACGCTCGGGTCCAGCTCCGCGAGCCAGGCGGGGATAGCACCACGACCGGTCGGTACGTCCTCTAC : 2754
AF131g00240        : GTCCAGGAATCGCATCGGTCATGCGAGCCACCCCAGGGCCTCTCGAAAGGGCAATACGCTCGGGTCCAGCTCCGCGAGCCGGGCGGGGATGGCACCACGACCGGTCGGTACGTCCTCTAC : 2643
aor8g00861(intron) : ------------------------------------------------------------------------------------------------------------------------ :    -
                     gtccaggaatcgcatcggtcatgcgagccaccccagggcctctcgaaagggcaatacgctcgggtccagctccgcgagcc ggcggggat gcaccacgaccggtcggtacgtcctctac

                              *      2780         *      2800         *      2820         *      2840         *      2860         *      2880
AF131g00218        : GTCCACGATATTCTGCCGCCGACACCGTCGCATGGTCCGGGTGCCCGTCTCGTCGTCACGAAATACTCCTTCCTGGCCGACCGGTTAGCATGGGAAGCCGGGCATCCAGACACTCAGGCC : 2874
AF131g00240        : GTCCACGATGTTCTGCCGCCGACCCCGTCGCATGGTTCGGGTGCCCGTCTCGTCGTCACGAAATACTCCTTCCTGGCCGACCGGTTAGCGTGGGAAGCCGGGCATCCAGACACTCAGGTC : 2763
aor8g00861(intron) : ------------------------------------------------------------------------------------------------------------------------ :    -
                     gtccacgat ttctgccgccgac ccgtcgcatggt cgggtgcccgtctcgtcgtcacgaaatactccttcctggccgaccggttagc tgggaagccgggcatccagacactcagg c

                              *      2900         *      2920         *      2940         *      2960         *      2980         *      3000
AF131g00218        : CAGGAGCTGCTCCTGCACTTCCGGGACGCTGCCCGGATTGGCTTTCCGGACGACGCGGAGATTATTAATGTCGACACCGTGGTCGCGGTGGATTCGATGGACACCGAGCCGGTTGGCCCG : 2994
AF131g00240        : CAGGAGCTGCTCCTGCACTTCCGGGACGCTGCCCGGATTGGCTATCCGGACGACGCGGAGATTATTAATGTCGACACCGTGGTCGCGGTGGATTCGATGGACACCGAGCCGGTTGGCCCG : 2883
aor8g00861(intron) : ------------------------------------------------------------------------------------------------------------------------ :    -
                     caggagctgctcctgcacttccgggacgctgcccggattggct tccggacgacgcggagattattaatgtcgacaccgtggtcgcggtggattcgatggacaccgagccggttggcccg

                              *      3020         *      3040         *      3060         *      3080         *      3100         *      3120
AF131g00218        : ACCGTGTCGTATGTCGCACAGGTGCCTGACCCGGCGAACGGCCTTTTCGTGCGGTATGGCATTTGTGAGGGATCCCAGCGGGAATGGTTGTTTCTGGCGCCGCTGGGGTCTTCGCTCACG : 3114
AF131g00240        : ACCGTGTCGTATGTCGCACAGGTGCCTGACCCGGCAAACGGCCTTTTCGTGCGGTATGGCATTCGTGAGGGGTCCCGACGGGAGTGGTTGTTTCTGGCGCCGCTGGTGTCTTCACTCACG : 3003
aor8g00861(intron) : ------------------------------------------------------------------------------------------------------------------------ :    -
                     accgtgtcgtatgtcgcacaggtgcctgacccggc aacggccttttcgtgcggtatggcatt gtgaggg tccc  cggga tggttgtttctggcgccgctgg gtcttc ctcacg

                              *      3140         *      3160         *      3180         *      3200         *      3220         *      3240
AF131g00218        : GGCCCGAGAGGCAGTGCCGCGTATCCACGTTTTTCTCCGCTGCCCGTGGCCTCCGTCTTCGATCTCTCCCCCGACGACCTGCGCCTGTCGGTTGGCTTCCAGAAGGCGGGATATTGCGTG : 3234
AF131g00240        : GGCCCGAGAGGCAGTGCCGCGTATCCACGCTTTTCACCGCTGCCCGTGGCCTCCGTCTTCGATCTCTCCCCCGACGACCTGCGCCTGTCGATTGGCTTCCAGAAGGCGGGATATTGCGTG : 3123
aor8g00861(intron) : ------------------------------------------------------------------------------------------------------------------------ :    -
                     ggcccgagaggcagtgccgcgtatccacg ttttc ccgctgcccgtggcctccgtcttcgatctctcccccgacgacctgcgcctgtcg ttggcttccagaaggcgggatattgcgtg

                              *      3260         *      3280         *      3300         *      3320         *      3340         *      3360
AF131g00218        : CAGGCCGCGGTTGGCTTTGCCGAAGACCGCCACCAGGCATGGAAGACGCAACATCCCGATGCCACCGTCTACCCGGGCTGTGTTGCATCCGCCCTCGCGACGATGGACTACGACCTCTCC : 3354
AF131g00240        : CAGGCCGCGGTCGGTTTTGCCGAAGACCGCCACCAGGCATGGAAGACGCAACATCCCGATGCCACCGTCTACCCGGGCTGTGTTGCATCCGCCCTCGCGACGATGGACTACGACCTCTCC : 3243
aor8g00861(intron) : ------------------------------------------------------------------------------------------------------------------------ :    -
                     caggccgcggt gg tttgccgaagaccgccaccaggcatggaagacgcaacatcccgatgccaccgtctacccgggctgtgttgcatccgccctcgcgacgatggactacgacctctcc
```

```
                               *       3380         *       3400         *       3420         *       3440         *       3460         *       3480
AF131g00218       : ACGCAACCGGATGACAGAGGGCGTGATGTGCCCCGCATTGTGACCATTAGTGGCGAGGGCTCTTTCGCTAAATCAGCCGCCGGTTGGCTCGACCCTAGCTCCTCGGGCTTCGATGGCTTC : 3474
AF131g00240       : ACGCAACCGGATGACAGAGGGCGTGATGTGCCCCGCATTGTGACCATTAGTGGCGAGGGCTCTTTCGCTAAATCAGCCGCCGGTTGGCTCGACCCTAGCTCCTCGGGCTTCGATGGCTTC : 3363
aor8g00861(intron): ------------------------------------------------------------------------------------------------------------------------ :    -
                    acgcaaccggatgacagagggcgtgatgtgccccgcattgtgaccattagtggcgagggctctttcgctaaatcagccgccggttggctcgaccctagctcctcgggcttcgatggcttc

                               *       3500         *       3520         *       3540         *       3560         *       3580         *       3600
AF131g00218       : GACAGTCTCTCCAGGGAGACGCTGAGTCCGCTTCAACAGTGTGAGCTCGCGGCGCAGTCTCCAGGGCTGAGTCCGGACTTCATCGTGCTGACGATGTCCCGGTCGATCTTGACAGACCCG : 3594
AF131g00240       : GACGGTCTCTCCGGGGAGAGGCTGAGTCCGCTTCAACAGTGCGAGCTCGCGGCGCAGTCTCCAGGGCTGAGTCCGGACTTCATCGTGCTGACGATGTCCCGGTCGATCTTGACAGACCAG : 3483
aor8g00861(intron): ------------------------------------------------------------------------------------------------------------------------ :    -
                    gac gtctctcc gggaga gctgagtccgcttcaacagtg gagctcgcggcgcagtctccagggctgagtccggacttcatcgtgctgacgatgtcccggtcgatcttgacagacc g

                               *       3620         *       3640         *       3660         *       3680         *       3700         *       3720
AF131g00218       : CGGTGGGGCTCACTGGCTCGCACCATGGGTCTTCTCCTCGAGCAAGGGTATGCGGTGGCGCTGCGGCCGGTCTCTCTGCCGGATCCGGGGCAGCATGGAGAGCGATCGGTTCTCCTCCTC : 3714
AF131g00240       : CGGTGGGGCTCACTGGCTCGCACCATGGGTCTTCTCCTCGAGCAAGGGTATGCGGTGGCGCTGCGGCCGGTCTCTCTGCCGGATCCGGGGCAGCATGGGGAGCGATCGGTTCTCCTCCTC : 3603
aor8g00861(intron): ------------------------------------------------------------------------------------------------------------------------ :    -
                    cggtggggctcactggctcgcaccatgggtcttctcctcgagcaagggtatgcggtggcgctgcggccggtctctctgccggatccggggcagcatgg gagcgatcggttctcctcctc

                               *       3740         *       3760         *       3780         *       3800         *       3820         *       3840
AF131g00218       : GCCGCCCCGGGCCGCACCGACCCACAGTGGATCGATGACACGCTTTCCGACCTGCGAATGGTATCAGCCTCGGACGAGCTGCTGGAACGTGCGGAGAACGAGAGCGGCGTCTCTAGCGTC : 3834
AF131g00240       : GCCGCCCCGGGCCGCACCGACCCACAGTGGATCGATGACACGCTTTCCGACCTGCGAATGGTATCAGCCTCGGACGAGCTGCTGGAACGTGCGGAGAACGAGAGCGGCGTCTCTAGCGTC : 3723
aor8g00861(intron): ------------------------------------------------------------------------------------------------------------------------ :    -
                    gccgccccgggccgcaccgacccacagtggatcgatgacacgctttccgacctgcgaatggtatcagcctcggacgagctgctggaacgtgcggagaacgagagcggcgtctctagcgtc

                               *       3860         *       3880         *       3900         *       3920         *       3940         *       3960
AF131g00218       : CCGGCCGCGGATGGCATAGCGGCAGCCGGGTCGGCGGATGTAGGCGTTGCTAGTCCTCCAGCGAATTCTACTCCTCGGCATGGTGACAGCGTTCTCGCGGATGGCGCACAACATATTACC : 3954
AF131g00240       : CCGGCCGCGGATGGCATAGCGGCAGCCGGGTCGGCGGATGTAGGCGATGCTAGTCCTCCAGCGAATTCTACTCCTCAGCATGGTGACAGCGTTCTCGCGGATGGGGCACAACATATTGCC : 3843
aor8g00861(intron): ------------------------------------------------------------------------------------------------------------------------ :    -
                    ccggccgcggatggcatagcggcagccgggtcggcggatgtaggcg tgctagtcctccagcgaattctactcctc gcatggtgacagcgttctcgcggatgg gcacaacatatt cc

                               *       3980         *       4000         *       4020         *       4040         *       4060         *       4080
AF131g00218       : GCCACGGTCTCCCGGATTATTGGAGAATTTTCAACCAAGTGCGCGCTGACGACGAACCTCCCCCGGTTGGTTGTGACCGACAATTCGGATGGCGCGCAGAACAGAGCCAAGCGGCAGAGG : 4074
AF131g00240       : GCCACGGTCTCCCGGATTATCGGAGAATTTTCAACCAAGTGCGCGCTGACGACGAACCTCCCCCGGTTGGTTGTGACCGACAATTCGGATGGCGCGCAGGACAGAGCCAAGCGACAGAGG : 3963
aor8g00861(intron): ------------------------------------------------------------------------------------------------------------------------ :    -
                    gccacggtctcccggattat ggagaattttcaaccaagtgcgcgctgacgacgaacctcccccggttggttgtgaccgacaattcggatggcgcgcag acagagccaagcg cagagg

AF131g00218       : ATGTAG : 4080
AF131g00240       : ATGTAG : 3969
aor8g00861(intron): ------ :    -
                    atgtag
```

d

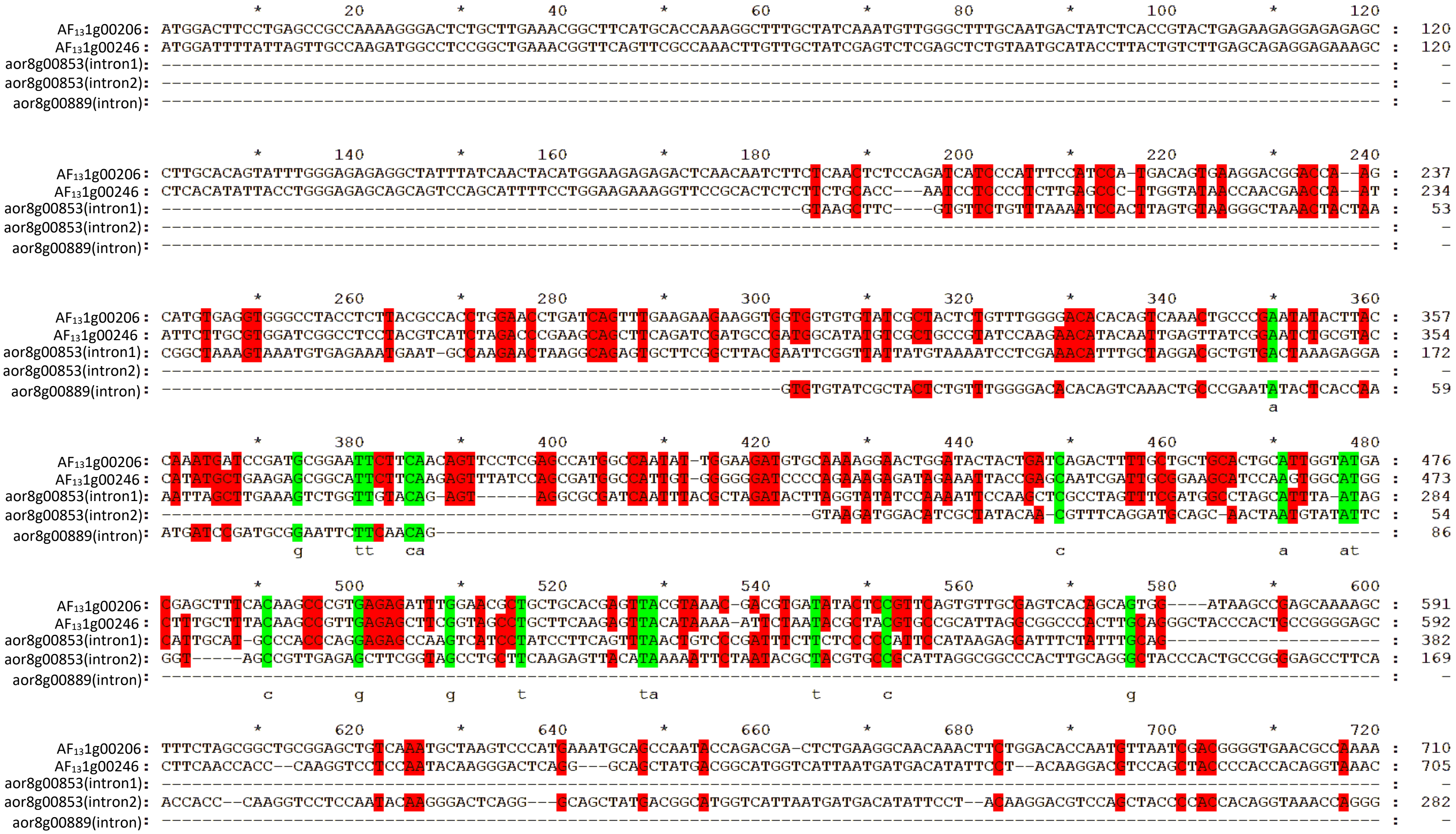
AF$_{13}$1g00206
AF$_{13}$1g00246
aor8g00853(intron1)
aor8g00853(intron2)
aor8g00889(intron)

```
                          *      620         *      640         *      660         *      680         *      700         *      720
AF13 1g00206        : TTTCTAGCGGCTGCGGAGCTGTCAAATGCTAAGTCCCATGAAATGCAGCCAATACCAGACGA-CTCTGAAGGCAACAAACTTCTGGACACCAATGTTAATCGACGGGGTGAACGCCAAAA :  710
AF13 1g00246        : CTTCAACCACC--CAAGGTCCTCCAATACAAGGGACTCAGG---GCAGCTATGACGGCATGGTCATTAATGATGACATATTCCT--ACAAGGACGTCCAGCTACCCCACCACAGGTAAAC :  705
aor8g00853(intron1) : ------------------------------------------------------------------------------------------------------------------------ :  -
aor8g00853(intron2) : ACCACC--CAAGGTCCTCCAATACAAGGGACTCAGG---GCAGCTATGACGGCATGGTCATTAATGATGACATATTCCT--ACAAGGACGTCCAGCTACCCCACCACAGGTAAACCAGGG :  282
aor8g00889(intron)  : ------------------------------------------------------------------------------------------------------------------------ :  -

                          *      740         *      760         *      780         *      800         *      820         *      840
AF13 1g00206        : TAGC-CTTAGCTGCCAGTCGCAACTCCCGCAGGATTTGGCAGAACGTATTTTGAATGAAAGCCCTCGGGCAAAGCAACTTTATTCATTGGAGCAACAGGGAACCGCGTCCAGTCATCAAT :  829
AF13 1g00246        : CAGGGTTTGGCTGAAAGGTCCTGCTCCCA---GATCCCCCAGA-CAGATCT-----GGATGTCGCGGAACAGCCCTGTGCTAC-CATTAGCTTTGGGGAGAAT----TCTCGCCACTGCT :  811
aor8g00853(intron1) : ------------------------------------------------------------------------------------------------------------------------ :  -
aor8g00853(intron2) : TTTGGCTGAAAGGTCCTGCTCCCA---GATCCCCCAGA-CAGATCT-----GGATGTCGCGGAACAGCCCTGTGCTAC-CATTAGCTTTGGGGAGAAT----TCTCACCACTGCTGTCCA :  388
aor8g00889(intron)  : ------------------------------------------------------------------------------------------------------------------------ :  -

                          *      860         *      880         *      900         *      920         *      940         *      960
AF13 1g00206        : TGGCTGAGGAGAATAACTTCTTTTCGAACCAGCTAAAAAGCCAGAAGGCAAATCCATCTAATGTTCACCGATGTTATATGCCAGGCTTCAAGTCGTATTCAGGGAAGATTCCGAGACAAA :  949
AF13 1g00246        : GTCTCAAAGAAGAGAAGGGCCTTTCGGACCAGCTAGAA----------------CATATACAGGTTGCTGAT-CCACAACCCAGAGAGCAGACCGGACACAAGAAAAGATGCAGAACAA- :  913
aor8g00853(intron1) : ------------------------------------------------------------------------------------------------------------------------ :  -
aor8g00853(intron2) : AAAGAAGAGAAGGGCCTTTCGGACCAGCTAGAACATATACAGGTTG-TTGATCTACAACCCAGAGA----GCAGACCGGACACAAGAAAAGATGCAGAACAAGAGGCCGAACAAAACGGA :  503
aor8g00889(intron)  : ------------------------------------------------------------------------------------------------------------------------ :  -

                          *      980         *     1000         *     1020         *     1040         *     1060         *     1080
AF13 1g00206        : AGAGGAAGAATAAGGTTGTGGCCAACAGTGGCA-GACATGG-AATGGCGAGCATTAACTGGTAG-------------------------------------------------------- : 1011
AF13 1g00246        : -GAGGCCGAACAGAACGGAGATTGACTGCTGCACGACGCGATAGCGCCAAGAGAAGGCAGACAACTTTCGCC------------------------------------------------ :  984
aor8g00853(intron1) : ------------------------------------------------------------------------------------------------------------------------ :  -
aor8g00853(intron2) : GATTGACTGCTGCACGACGCGATAGCGCCAAGAGAAGGCAGACAACTTTCGCCCGTACGTAACGTACACAGAACGTTGAACAGACCCTTGCATCAATCTTAAGTACATTATCCAGAAAGT :  623
aor8g00889(intron)  : ------------------------------------------------------------------------------------------------------------------------ :  -

                          *     1100         *     1120         *     1140         *     1160
AF13 1g00206        : -------------------------------------------------------------------------------- :  -
AF13 1g00246        : -------------------------------------------------------------------------------- :  -
aor8g00853(intron1) : -------------------------------------------------------------------------------- :  -
aor8g00853(intron2) : TTATTGTCAGTCACCCGATTGCTCGCCTAAGTGCTCATATGACCCACAATGACTTTTAGGCCTGTACGTACACAAGGCTTAG :  705
aor8g00889(intron)  : -------------------------------------------------------------------------------- :  -
```

Supplemental Figure S3. Highly mutated regions identified flanking the converted regions. a. For the homologous gene group 1, a highly dissimilar region contains 46 base pairs with mere 18 identical ones; b. For the homologous gene group 2, three highly dissimilar regions were identified between converted regions, with the first containing 140 base pairs with 56 identical ones (b1); the second 7 base pairs with 1 identical base pair (b2), and the third contains 55 base pairs with 19 identical ones (b3).

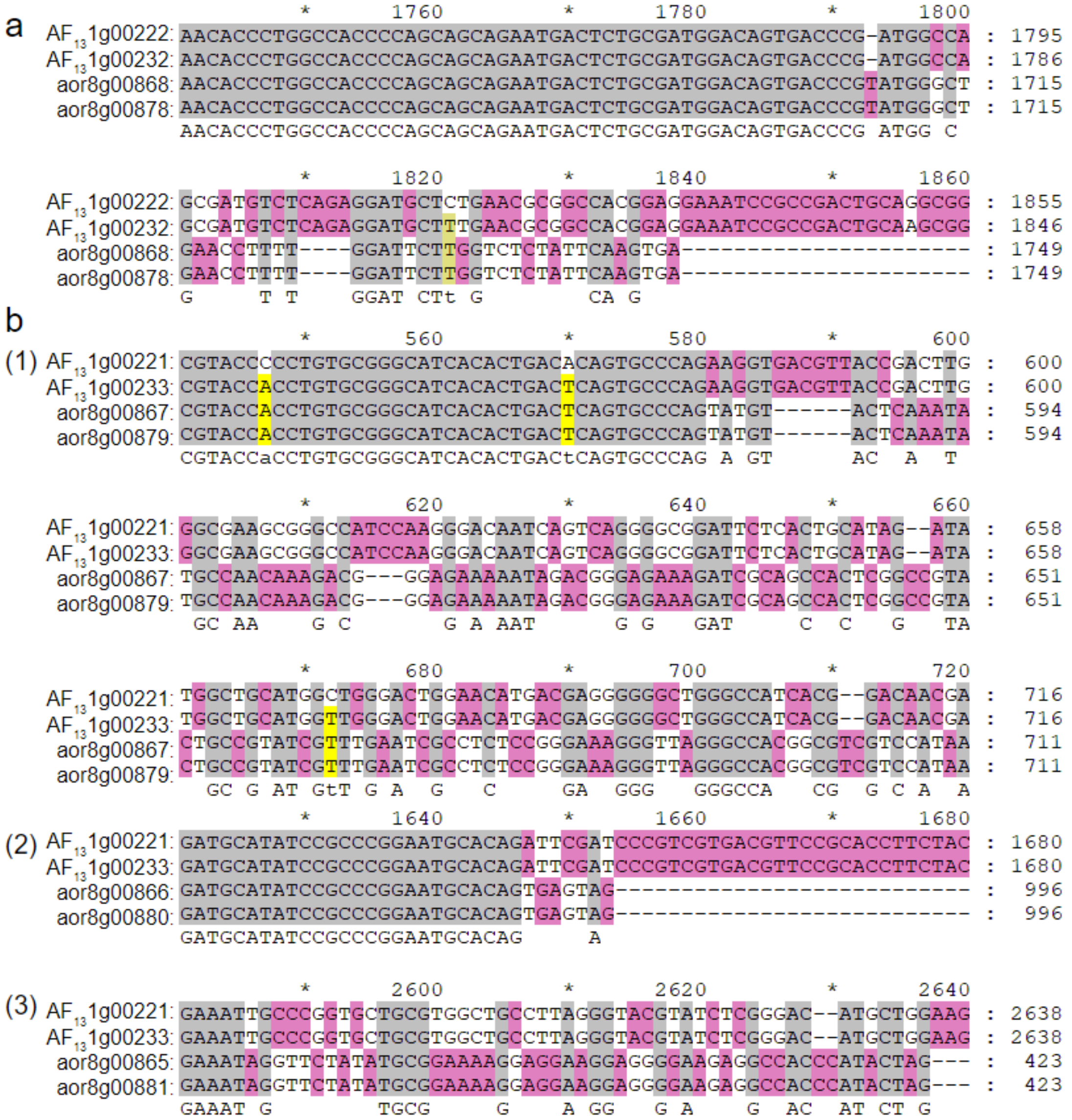

Supplement: Supplementary file 1 [file microorganisms-14-00033-s001.zip › Supplemental Figures.pdf]
